# Molecular Characterization of Penicillin-Binding Protein2x, 2b and 1a of *Streptococcus pneumoniae* Causing Invasive Pneumococcal Diseases in China: A Multicenter Study

**DOI:** 10.3389/fmicb.2022.838790

**Published:** 2022-03-01

**Authors:** Menglan Zhou, Lulu Wang, Ziran Wang, Timothy Kudinha, Yao Wang, Yingchun Xu, Zhengyin Liu

**Affiliations:** ^1^Department of Clinical Laboratory, State Key Laboratory of Complex Severe and Rare Diseases, Peking Union Medical College Hospital, Chinese Academy of Medical Sciences and Peking Union Medical College, Beijing, China; ^2^Beijing Key Laboratory for Mechanisms Research and Precision Diagnosis of Invasive Fungal Diseases, Beijing, China; ^3^Nanjing Hospital of Chinese Medicine Affiliated to Nanjing University of Chinese Medicine, Nanjing, China; ^4^School of Biomedical Sciences, Charles Sturt University, Orange, NSW, Australia; ^5^NSW Health Pathology, Regional and Rural, Orange Hospital, Orange, NSW, Australia; ^6^Department of Infectious Disease, Peking Union Medical College Hospital, Chinese Academy of Medical Sciences, Beijing, China

**Keywords:** *Streptococcus pneumoniae*, penicillin susceptibility, penicillin-binding protein, serotype, invasive pneumococcal disease

## Abstract

*Streptococcus pneumoniae* is a common human pathogen that can cause severe invasive pneumococcal diseases (IPDs). Penicillin-binding proteins (PBPs) are the targets for β-lactam antibiotics (BLAs), which are the common empirical drugs for treatment of pneumococcal infection. This study investigated the serotype distribution and antibiotic resistance patterns of *S. pneumoniae* strains causing IPD in China, including exploring the association between penicillin (PEN) susceptibility and PBPs variations. A total of 300 invasive *S. pneumoniae* isolates were collected from 27 teaching hospitals in China (2010-2015). Serotypes were determined by Quellung reaction. Serotypes 23F and 19F were the commonest serotypes in isolates from cerebrospinal fluid (CSF), whilst serotypes 19A and 23F were most commonly seen in non-CSF specimens. Among the 300 invasive *S. pneumoniae* strains, only one strain (serotype 6A, MIC = 0.25 μg/ml) with PEN MIC value ≤ 0.25 μg/ml did not have any substitutions in the PBPs active sites. All the strains with PEN MIC value ≥ 0.5 μg/ml had different substitutions within PBPs active sites. Substitutions in PBP2b and PBP2x active sites were common in low-level penicillin-resistant *S. pneumoniae* (PRSP) strains (MIC = 0.5 μg/ml), with or without PBP1a substitution, while all strains with PEN MIC ≥ 1 μg/ml had substitutions in PBP1a active sites, accompanied by PBP2b and PBP2x active site substitutions. Based on the three PBPs substitution combinations, a high degree of diversity was observed amongst the isolates. This study provides some new insights for understanding the serology and antibiotic resistance dynamics of *S. pneumoniae* causing IPD in China. However, further genomic studies are needed to facilitate a comprehensive understanding of antibiotic resistance mechanisms of *S. pneumoniae.*

## Introduction

*Streptococcus pneumoniae* is one of the most common Gram-positive cocci that is mainly transmitted through the respiratory tract. The organism usually colonizes the human nasopharynx and can migrate to the middle ear and lungs causing local non-invasive pneumococcal disease (NIPD) such as otitis media and pneumonia in immune-deficient people (Lynch and Zhanel, 2009; Henriques-Normark and Tuomanen, 2013). Data from the World Health Organization (WHO) shows that pneumonia killed 808, 694 children under five years old in 2017, accounting for 15% of all deaths in children. *S. pneumoniae* is the most common pneumonia pathogen in children worldwide, with a mortality rate in children much higher than other diseases such as AIDS, malaria, and measles (Lynch and Zhanel, 2009). In Europe and America, *S. pneumoniae* is also the most common cause of community-acquired pneumonia in adults (World Health Organization [WHO], 2007). In addition to respiratory tract infections, *S. pneumoniae* can also migrate to the blood and brain and cause severe invasive pneumococcal disease (IPD), such as bacteremia, meningitis *etc*., causing a huge economic and medical burden on both developed and developing countries (Mehr and Wood, 2012).

The major empirical antimicrobial drugs used in the treatment of *S. pneumoniae* infections are β-lactam antibiotics (BLAs), which act on the bacterial cell wall. Penicillin-binding proteins (PBPs) are crucial enzymes in the biosynthesis of peptidoglycan (PG), a major cell wall component that surrounds the cytoplasmic membrane and is required to maintain the shape and osmotic stability of bacteria (Hakenbeck et al., 2012). The target of BLAs are PBPs, and function by covalently binding to the active site serine of PBPs through the β-lactam ring, thereby interfering with the synthesis of bacterial cell walls and eventually leading to bacterial cell death. With the widespread use of antibiotics, penicillin-intermediate *S. pneumoniae* (PISP) and penicillin-resistant *S. pneumoniae* (PRSP), commonly referred to as penicillin-non-susceptible *S. pneumoniae* (PNSP), have emerged and are detected continually worldwide. Data from the Asian Network for Surveillance of Resistant Pathogens (ANSORP) shows that the isolation rate of PNSP from 2012 to 2017 (9.0%) was significantly higher than that from 2008 to 2009 (4.9%), and the detection rates of PNSP from patients in China was 1.9% (2012-2017, oral breakpoint) (Kim et al., 2012, 2020).

The main mechanism of BLAs resistance by *S. pneumoniae* is through PBPs substitutions. Alterations in PBPs via substitutions reduce their reactivity for β-lactam attachment to the binding site and thereby reduce their effectiveness (Zapun et al., 2008). *S. pneumoniae* has six PBPs, but only three PBPs, PBP2x, PBP2b and PBP1a play a main role in BLAs resistance. Alterations in all other PBPs have been described occasionally (Zapun et al., 2008). Mosaicity is the product of homologous recombination that causes the sequence diversity in *S. pneumoniae*. In most resistant clinical *S. pneumoniae* isolates, the sequencing revealed that the mosaic genes encode PBP2x, PBP2b, and PBP1a (Laible et al., 1991; Martin et al., 1992; Sibold et al., 1994). The active sites of PBPs comprises three conserved sequences SXXK, SXN and KT(S)G. The serine of the SXXK motif is the active site residue that reacts with BLAs. They are located in PBP2x: 337STMK340, 395SSN397, 547KTG549; in PBP2b: 386STMK389, 443SSN445, and 615KTG617; and in PBP1a: 370STMK373, 428SRN430, 557KTG559 (Hakenbeck et al., 2012). Changes in these active site motifs and their adjacent sequences lead to a decrease in the affinity of PBPs to penicillin resulting in antibiotic resistance (Zhanel et al., 2006; Hakenbeck et al., 2012). Few studies have been carried out to understand the association between penicillin susceptibility and PBPs variations in *S. pneumoniae* isolates from patients with IPD in mainland China.

Due to the widespread use of antimicrobial drugs, and the fastidious nature of *S. pneumoniae*, the number of isolates from IPD specimens is very low, and hence a scarcity of relevant research studies (Xue et al., 2010; Yao et al., 2011; Quan-Cheng et al., 2016). This study aimed to analyze the serotype distribution and antibiotic resistance pattern of *S. pneumoniae* strains causing IPD in China, and to explore the association between penicillin susceptibility and PBPs variations.

## Materials and Methods

### Bacterial Isolates

A total of 300 non-duplicate invasive *S. pneumoniae* isolates from 27 teaching hospitals in 13 provinces of China (2010-2015) were studied (Supplementary Table 1). The isolates were transported to the Department of Clinical Laboratory in Peking Union Medical College Hospital for re-identification and further analysis. The most common specimen type was blood, accounting for 72.7% (218/300) of the isolates, followed by cerebrospinal fluid (CSF) (19.0%, 57/300) and pleural effusion (5.7%, 17/300). Other specimen types included ascites, joint drainages, pleural drainage and lung tissue, each accounting for ≤ 2.0% of the isolates. The majority of the patients were males, accounting for 65.7% (197/300) of the isolates. The average age of patients was 46 ± 26.69 years old.

### Serotyping

All the *S. pneumoniae* isolates were serotyped by Quellung reaction (Maha et al., 2014). The serotype was first determined by latex agglutination test using the checkerboard typing system. Then specific type antiserum was mixed with the bacterial suspension to determine the final serotype. Capsular swelling was observed under oil immersion microscope. If the test strain was negative with all antisera, it was classified as non-typeable (NT).

### Antimicrobial Susceptibility Testing

The minimum inhibitory concentrations (MICs) of *S. pneumoniae* against penicillin (PEN), amoxicillin/clavulanic (AMC), cefuroxime (CXM), ceftriaxone (CRO), cefepime (FEP), ertapenem (ETP), imipenem (IPM), meropenem (MEM), levofloxacin (LEV), trimethoprim/sulfamethoxazole (SXT), clindamycin (DA), clarithromycin (CLA), erythromycin (E), linezolid (LZD) and vancomycin (VA), were determined by broth microdilution method as recommended by the Clinical and Laboratory Standards Institute (CLSI) M07-A10 (Clinical and Laboratory Standards Institute (CLSI), 2012). *S. pneumoniae* ATCC49619 and *Escherichia coli* ATCC25922 were used as the quality control strains, and were tested along each batch. Results were considered valid when the MIC values of the quality control strains were within the expected range. Antimicrobial susceptibility testing results were interpreted according to CLSI 2019 guidelines (Clinical and Laboratory Standards Institute (CLSI), 2019).

### Penicillin-Binding Protein Gene Amplification and Sequencing

The isolates were cultured on blood agar plates and incubated overnight at 35°C in a 5% CO_2_ atmosphere. DNA was extracted using the AxyGen amp DNA Mini Extraction Kit (Axygen, United States) according to the manufacturer’s instructions. The final pure DNA was stored at −20°C until use. The nucleotide sequence of an around1-kb region encoding the penicillin-binding domain of *pbp2x, pbp2b* and *pbp1a* genes were amplified and sequenced based on published primers (Table 1; Zhanel et al., 2006). PCR products were sent to Beijing RuiBiotech Co., Ltd., for sequencing.

**TABLE 1:** Primers for amplification and sequencing for the region encoding the penicillin-binding domain of *pbp2x*, *pbp2b* and *pbp1a* genes.

Gene name	Primer name	Primer sequences (5′-3′)	Target fragment length (bp)
*pbp2x*	F	TATGAAAAGGATCGTCTGGG or	1148
		TATGAAAAAGACCGTGTAGG	
	R	AGAGAGTCTTTCATAGCTGAAGC	
*pbp2b*	F	GGCTATTCTCTAAATGACCGT	1317
	R	AGCTTAGCAATAGGTGTTGG	
*pbp1a*	F	TGGGATGGATGTTTACACAAATG	1197
	R	GTCGTACTATTATTTGTGCTTGG	

CLC Sequence Viewer software (CLC, Denmark) was used to manually correct the sequencing peaks and sequences to ensure the sequencing quality, and a two-way splicing was carried out. The spliced sequences were translated to simulated protein sequences and compared with PBP2x, PBP2b, and PBP1a corresponding to *S. pneumoniae* reference strain R6 (PSSP, GenBank accession No. NC003098) for variation analysis.

### Data Analysis and Statistical Analysis

Differences in antimicrobial susceptibility were analyzed by MIC range, MIC_50_ and MIC_90_, and statistical analysis was performed by chi-square test or Fisher’s exact probability test using SPSS software (version 22.0, SPSS Inc., Chicago, IL, United States). *P* value < 0.05 was considered statistically significant.

## Results

### Serotype Distribution

Based on the Quellung reaction, a total of 299 *S. pneumoniae* isolates were identified to the serotype level accurately and one strain was considered as non-typeable (NT). Among the 299 serotypeable isolates, 41 serotypes were detected. The top five serotypes were: 23F (14.3%, 43/300), 19F (13.7%, 41/300), 19A (13.7%, 41/300), 3 (10.3%, 31/300) and 14 (9.0%, 27/300). The main serotypes in CSF isolates were 23F and 19F, both accounting for 15.8% (9/57) each, whilst in non-CSF specimens, serotypes 19A and 23F were the most common, accounting for 14.8% (36/243) and 14.0% (34/243) of the isolates, respectively (Table 2).

**TABLE 2:** Serotype distribution of 300 invasive *S. pneumoniae* isolates in different specimens.

Serotype	CSF		Non-CSF		Total percentage (%)
	No.	Percentage (%)	No.	Percentage (%)	
23F	9	15.8	34	14.0	14.3
19F	9	15.8	32	13.2	13.7
19A	5	8.8	36	14.8	13.7
3	4	7.0	27	11.1	10.3
14	7	12.3	20	8.2	9.0
6A	3	5.3	9	3.7	4.0
6B	1	1.8	10	4.1	3.7
Others	19	33.3	75	30.9	31.3
Total	57	100	243	100	100

### Antimicrobial Susceptibility

Concerning non-BLAs drugs, all the *S. pneumoniae* isolates were susceptible to LEV, VA and LZD, with MIC_90_ values of 1 μg/ml, 0.5 μg/ml, and 1 μg/ml. The prevalence of resistance of the isolates to SXT was 65.3%. Resistance rates to CLA, E and DA were extremely high, all above 90%. PISP accounted for 4.3% of the isolates based on the non-meningitis (R ≥ 8 μg/ml) breakpoint while none, 67.7% and 44.7% of the isolates were classified as PRSP based on non-meningitis, meningitis (R ≥ 0.12 μg/ml) and oral administration (R ≥ 2 μg/ml) breakpoints, respectively. Susceptibility to AMC was about 97.7%. The prevalence of resistance of the isolates to the second-generation cephalosporins CXM was about ≥60%. The third- and fourth-generation cephalosporins CRO and FEP had the same MIC_90_ value of 2 μg/ml. The resistance rates of the isolates to IPM and MEM was only 3.7 and 2.7%, respectively, but the intermediate rates were much higher at 37.7 and 46.0%, respectively (Table 3).

**TABLE 3:** Antimicrobial susceptibility results of 300 *S. pneumoniae* isolates.

Antimicrobial agent	Breakpoint type	%R	%I	%S	MIC_50_ (μg/ml)	MIC_90_ (μg/ml)	MIC range (μg/ml)
PEN	non-meningitis	0	4.3	95.7	1	2	≤ 0.015 - 4
PEN	meningitis	67.7	0	32.3	1	2	≤ 0.015 - 4
PEN	oral	44.7	23	32.3	1	2	≤ 0.015 - 4
MC	non-meningitis	0.3	2	97.7	0.5	2	0.015 - 8
CXM	parenteral	64.3	2	33.7	4	16	0.06 - 64
CXM	oral	60	4.3	35.7	4	16	0.06 - 64
CRO	non-meningitis	7.3	18.3	74.3	0.5	2	0.007 - 8
CRO	meningitis	25.7	23.3	51	0.5	2	0.007 - 8
FEP	non-meningitis	4	25.7	70.3	1	2	0.03 - 8
FEP	meningitis	29.7	33	37.3	1	2	0.03 - 8
ETP		0	0	100	0.125	0.25	0.004 - 1
IPM		3.7	37.7	58.7	0.125	0.25	0.008 - 8
MEM		2.7	46	51.3	0.25	0.5	0.004 - 1
LEV		0	0	100	0.5	1	0.007 - 2
SXT		65.3	13	21.7	8	16	0.12 - 128
DA		95.7	1.7	2.7	128	128	0.12 - 256
CLA		96	0	4	> 1024	> 1024	0.06 - 2048
E		96	0	4	> 1024	> 1024	0.06 - 2048
VA		0	0	100	0.5	0.5	0.015 - 1
LZD		0	0	100	1	1	0.5 - 1

*PEN, penicillin; AMC, Amoxicillin-clavulanic acid; CXM, cefuroxime; CRO, ceftriaxone; FEP, cefepime; ETP, ertapenem; IPM, imipenem; MEM, meropenem; LEV, levofloxacin; SXT, trimethoprim-sulfamethoxazole; DA, clindamycin; CLA, clarithromycin; E, erythromycin; VA, vancomycin; LZD, Linezolid.*

### Association Between Serotypes, Penicillin Susceptibility and Variations in Penicillin-Binding Proteins Active Sites

Among the 300 isolates studied, 106 isolates, including all strains of serotypes1-5, 6C, 7F, 9A, 9N, 9V, 8, 17, 34, 10A, 11A, 12F, 15F, 17A, 18C, 24F, 25A, 25F, 28A, and 28F, one strain each of serotypes 6A, 7C, 19A and 23F, and two strains each of serotypes 6B, 13, 29, and 15A, exhibited PEN MIC values of ≤0.25 μg/ml. None of these isolates had PBPs substitution in the three conserved motifs and nearby sites, except one isolate of serotype 6A (PEN MIC = 0.25 μg/ml), in which TAA6A substitution was detected in the active sites of PBP2b. The remaining 194 isolates all had different amino acid substitutions in the PBPs active sites. In the PBP2x conserved motifs or nearby sites, 181 isolates had T338A substitution (threonine → alanine), 175 isolates had L546V substitution (leucine → valine), and 39 isolates had M339F substitution (methionine → phenylalanine), 4 isolates had H394L substitution (histidine → leucine), and the substitution rates were 60.3, 58.3, 13, and 1.3%, respectively. In the PBP2b conserved motifs or nearby sites, 190 isolates had T446A substitution (threonine → alanine), 3 isolates had T446S substitution (threonine → serine), and 72 isolates had A619G substitution (alanine → glycine), with substitution rates of 63.3, 1, and 24%, respectively. In the PBP1a conserved motifs or nearby sites, 53 isolates had T371A substitution (threonine → alanine), 122 isolates had T371S substitution (threonine → serine), and 175 isolates had P432T substitution (proline → threonine), with substitution rates of 17.7, 40.7, and 58.3%, respectively (Table 4).

**TABLE 4:** Association of serotypes, penicillin susceptibility and variations of conserved motifs forming or surrounding active penicillin-binding proteins (PBPs) binding sites in PBP2x, PBP2b, PBP1a among 300 *S. pneumoniae* isolates.

Serotype	MIC (μg/ml)	No.		PBP2x					PBP2b		PBP1a	
				T338	M339	H394	L546		T446	A619	T371	P432
1	≤ 0.015	5										
2	≤ 0.015	1										
3	≤ 0.015-0.03	31										
4	≤ 0.015	1										
5	≤ 0.015	2										
6A	0.25	1							A			
6B	0.25	2										
6C	≤ 0.015-0.25	2										
7C	0.03	1										
7F	≤ 0.015	2										
9A	≤ 0.015	2										
9N	≤ 0.015	2										
9V	≤ 0.015	3										
8	≤ 0.015	4										
13	≤ 0.015-0.12	2										
17	≤ 0.015	1										
20	≤ 0.015	3										
29	≤ 0.015-0.03	2										
34	≤ 0.015-0.03	7										
10A	≤ 0.015-0.06	3										
11A	≤ 0.015	4										
12F	≤ 0.015	4										
15A	≤ 0.015	2										
15F	≤ 0.015-0.25	2										
17A	≤ 0.015	1										
18C	≤ 0.015	1										
19A	≤ 0.015	1										
23F	≤ 0.015	1										
24F	≤ 0.015	3										
25A	0.12-0.25	2										
25F	0.25	1										
28A	≤ 0.015	2										
28F	≤ 0.015	3										
33B	0.03	2										
6A	0.5	1		A			V		A			T
6A	0.5	1		A			V					
6A	1	1		A			V		A	G	A	T
6A	2	8		A			V		A		A	T
6B	0.5	1		A	F		V		A		A	T
6B	0.5	1		A			V		A		A	T
6B	0.5	2		A			V		A		S	T
6B	0.5	1		A			V		S		S	T
6B	0.5	1		A					A			
6B	0.5	1				L						
6B	1	2		A			V		A		A	T
7C	2	1		A			V		A		A	T
13	0.5	1		A					A			
14	0.5	1		A			V		A		A	T
14	0.5	1				L			A		S	
14	0.5	1		A					A			
14	1	2		A			V		A		A	T
14	1	1		A			V		A		S	T
14	2	20		A			V		A		S	T
14	4	1		A			V		A		S	T
20	0.5	4		A					A			
29	0.5	1				L	V		A			
15A	0.5	1							A			
15B	1	1		A			V		A	G	S	T
15B	1	1		A			V		A		S	T
15B	1	1		A			V		A		S	T
15B	1	1		A			V		A	G		
15C	1	6		A			V		A		S	T
19A	2	5		A	F		V		A	G	S	T
19A	2	28		A			V		A	G	S	T
19A	2	1							A	G	S	T
19A	4	6		A			V		A	G	S	T
19F	0.5	2		A			V		A		S	T
19F	0.5	1							A			
19F	1	1		A	F		V		A		A	T
19F	1	8		A			V		A		S	T
19F	2	24		A	F		V		A	G	S	T
19F	4	3		A	F		V		A	G	S	T
19F	4	1		A			V		A	G	S	T
19F	4	1		A	F		V		S	G	S	T
22F	0.5	1							A			
23A	0.5	1							A		A	T
23A	0.5	1				L			A			
23A	1	2		A			V		A		A	T
23F	0.5	3							A			
23F	1	1		A	F		V		A		S	T
23F	1	3		A			V		A		S	T
23F	2	3		A	F		V		A		A	T
23F	2	27		A			V		A		A	T
23F	2	1							A		A	T
23F	2	3		A			V		A		S	T
23F	4	1		A			V		A		A	T
NT	1	1		A			V		S		S	T
Total	–	300	181	39	4	175	A/S: 190/3	72	A/S: 53/122	175		
Mutation rate (%)	–	–	60.3	13	1.3	58.3	A/S: 63.3/1	24	A/S: 17.7/40.7	58.3		

Based on the serotype and PEN MIC distribution among the isolates, ten isolates had only one PBP gene active site or nearby sites substitution, including two isolates each of serotype 6A and 6B, one isolate each of serotypes 19F, 22 F and 23A, and three isolates of serotype 23F. Seven of these isolates had PBP2b active sites or nearby sites substitutions; two had only PBP2x active sites or nearby sites substitutions, while none had any substitution in single PBP1a active sites or nearby sites. Save for one isolate of serotype 6A with PEN MIC of 0.25 μg/ml, all these strains had an MIC of 0.5 μg/ml. Thirteen isolates had substitutions in two of the PBPs gene active sites or nearby sites, including four isolates of serotype 20, two isolates of serotype 23A, and one isolate each for serotypes 6B, 13, 14, 29, 15B, 19A, and 23F. Among them, three isolates had substitutions in both PBP2b and PBP1a genes (serotypes 19A, 23F, and 23A), and the rest had substitution in PBP2x and PBP2b. The PEN MIC of most isolates in the group with two substitutions in the PBPs gene active sites or nearby sites was 0.5 μg/ml. The remaining 171 isolates had substitutions in the active sites or nearby sites of all the three PBPs genes, mainly distributed in serotypes 14 (*n* = 26), 19A (*n* = 39), 19F (*n* = 40) and 23F (*n* = 38).

Taken together, all isolates of serotypes 1, 2, 3, 4, 5, 6C, 7F, 9A, 9N, 9V, 8, 17, 34, 10A, 11A, 12F, 15F, 17A, 18C, 24F, 25A, 25F, 28A, 28F, and 33B, had no amino acid changes in the three active sites or nearby sites of PBPs (MIC ≤ 0.25 μg/ml), and all isolates of serotypes 14, 15B, 15C, 19F, 22F, and 23A, had at least one substitution in the active sites or nearby sites of PBPs (0.5 μg/ml ≤ MIC ≤ 4 μg/ml).

### Association Between Specimen Types and Variations in Penicillin-Binding Proteins Active Sites

**FIGURE 1:**
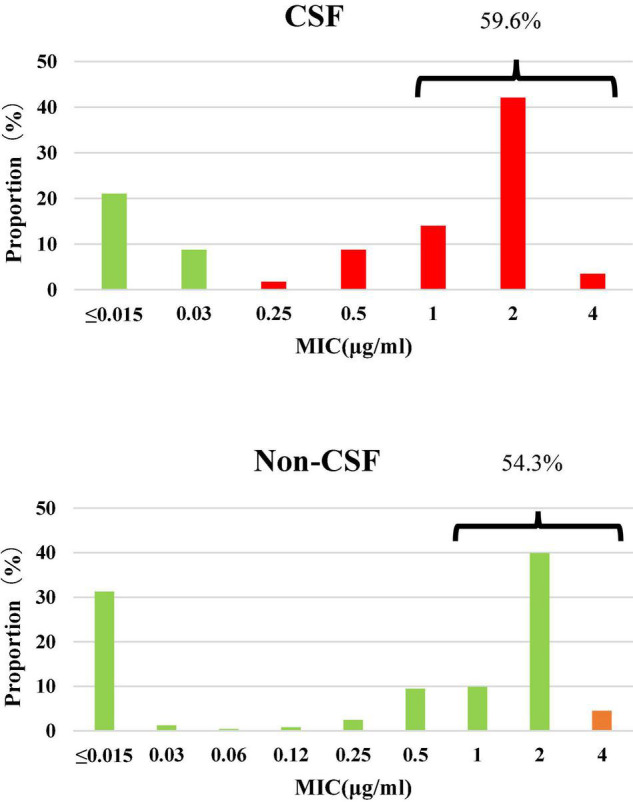
Susceptibility and MIC distribution against penicillin in 300 *S. pneumoniae* isolates. Green: susceptible, orange: intermediate, red: resistant. CSF: PSSP 29.8%, PISP 0%, PRSP 70.2%; Non-CSF: PSSP 95.5%, PISP 4.5%, PRSP 0%.

Among the 300 invasive *S. pneumoniae* isolates, 57 (19.0%) were isolated from CSF, and the rest (81.0%) from other sterile body fluids, mostly in blood. Antimicrobial susceptibilities were interpreted according to meningitis and non-meningitis break points. PRSP and PSSP from CSF accounted for 70.2 and 29.8% of the isolates, respectively. PISP and PSSP from non-CSF accounted for 4.5 and 95.5% of the isolates, respectively. Although the proportion of PRSP derived from CSF was significantly higher than that from non-CSF sources (*P* < 0.0001), the proportion of CSF derived isolates with PEN MIC ≥ 1μg/ml (59.6%) was similar to that from non-CSF sources (54.3%) (*P* = 0.5644) (Figure 1).

Analysis of PBPs active sites of strains from CSF showed that all PSSP, and one strain of PRSP, with PEN MIC of 0.25 μg/ml, had no substitution in the PBPs active sites. PBP2x and PBP2b substitutions were common in low-level PRSP strains (MIC = 0.5μg/ml), with or without PBP1a substitution. The strains with PEN MIC ≥ 1 μg/ml all had substitutions in the PBP1a active site or nearby sites, accompanied by substitutions in the PBP2x and PBP2b regions (Table 5).

**TABLE 5:** Association of penicillin susceptibility and variations of conserved motifs forming or surrounding active penicillin-binding proteins (PBPs) binding sites in PBP2x, PBP2b and PBP1a in *S. pneumoniae* isolates from CSF.

SIR	MIC (μg/ml)	No.	PBP2x				PBP2b		PBP1a	
			T338	M339	H394	L546	T446	A619	T371	P432
PSSP	≤ 0.03	17								
PRSP	0.25	1								
	0.5	1				A		A	T	
	0.5	2	A		V	A		S	T	
	0.5	2	A			A				
	1	1	A		V	A	G	A	T	
	1	1	A		V	A	G	S	T	
	1	6	A		V	A		S	T	
	2	2	A	F	V	A		A	T	
	2	6	A		V	A		A	T	
	2	6	A	F	V	A	G	S	T	
	2	3	A		V	A	G	S	T	
	2	7	A		V	A		S	T	
	4	1	A		V	A		A	T	
	4	1	A		V	A	G	S	T	

*PSSP, penicillin-susceptible S. pneumoniae; PISP, penicillin-intermediate S. pneumoniae; PRSP, penicillin-resistant S. pneumoniae.*

Analysis of PBPs active sites or nearby sites of strains derived from non-CSF sources revealed that all PSSP with PEN MIC ≤ 0.25 μg/ml had no substitutions in PBPs active sites or nearby sites, except one serotype 6A strain, in which a T446A substitution nearby the active site of PBP2b was detected. Various substitutions in the PBPs active sites or nearby sites of PSSP strains with PEN MIC ≥ 0.25 μg/ml were detected. Similar to CSF derived strains, the substitutions in active sites or nearby sites of PBP2x and PBP2b were common in strains with low PEN MIC level, with or without PBP1a active site substitution. In contrast, strains with PEN MIC ≥ 1 μg/ml all had substitutions in PBP1a active sites or nearby sites, accompanied by PBP2x or PBP2b substitutions (Table 6).

**TABLE 6:** Association of penicillin susceptibility and variations of conserved motifs forming or surrounding active penicillin-binding proteins (PBPs) binding sites in PBP2x, PBP2b, PBP1a in *S. pneumoniae* isolates from non-CSF specimens.

SIR	MIC (μg/ml)	No.	PBP2x				PBP2b		PBP1a	
			T338	M339	H394	L546	T446	A619	T371	P432
	≤ 0.12	82								
	0.25	1					A			
	0.25	5								
PSSP	0.5	1	A	F		V	A		A	T
	0.5	5	A			V	A		A	T
	0.5	1			L		A		S	
	0.5	1	A			V	A			T
	0.5	5	A				A			
	0.5	1			L	V	A			
	0.5	1			L		A			
	0.5	6					A			
	0.5	1	A			V				
	0.5	1			L					
	1	1	A	F		V	A		A	T
	1	6	A			V	A		A	T
	1	1	A	F		V	A		S	T
	1	15	A			V	A		S	T
	1	1	A			V	A	G		
	2	1	A	F		V	A		A	T
	2	30	A			V	A		A	T
	2	1					A		A	T
	2	23	A	F		V	A	G	S	T
	2	25	A			V	A	G	S	T
	2	1					A	G	S	T
	2	16	A			V	A		S	T
	4	3	A	F		V	A	G	S	T
	4	6	A			V	A	G	S	T
PISP	4	1	A			V	A		S	T
	4	1	A	F		V	S	G	S	T

*PSSP, penicillin-susceptible S. pneumoniae; PISP, penicillin-intermediate S. pneumoniae; PRSP, penicillin-resistant S. pneumoniae.*

### Variations of the *pbp2x* Gene

Compared with the reference strain R6 (GenBank accession No. NC003098), a total of 338 amino acids from positions 259 to 596 of the PBP2x protein were analyzed. A total of 98 different substitutions at 73 amino acid positions were detected in the PBP2x sequence, among which D567N (Asp → Asn) was the most common substitution, accounting for 69.3% (208/300), followed by D488N (Asp → Asn) and S576N (Ser → Asn), each accounting for 64.0% (192/300). Strains with L565S (Leu → Ser) and R384G (Arg → Gly) substitutions each accounted for 62.7% (188/300) and 61.3% (184/300), respectively. According to the distribution of various substitutions in PBP2x, all strains could be divided into 43 groups. The number of substitution sites in each group ranged from 1 to 42, accounting for 0-12.4% of the total number of amino acids analyzed. Group 2X37 was the commonest, accounting for 16.3% of all strains (49/300). There were 38 substitutions detected in all strains within the group, and the substitution rate was 11.2%. The second common group was 2X01, accounting for 13.3% of all strains (40/300), and the PBP2x sequence of strains in this group was exactly the same as that of R6 strain. There were 33 strains in the 2X41 group, accounting for 11.0% of the strains. Forty substitutions were detected in all strains of this group and the substitution rate was 11.8% (Table 7). Compared with PBP2b and PBP1a sequences, PBP2x had the least number of total substitutions (98 vs. 112 vs. 105). According to the distribution of PEN MIC, the number of detectable substitution sites in strains with PEN MIC ≤ 0.25 μg/ml ranged from 10-32, while for strains with PEN MIC = 0.5, 1, 2, and 4 μg/ml the number of detectable substitutions were 86, 46, 57, and 48, respectively (Supplementary Table 2).

**TABLE 7:** Deduced amino acid sequences of PBP2x in 300 *S. pneumoniae* isolates.

PBP2x group	N	Percent (%)	Number of substitution sites	Substitution rate (%)	2	2	2	2	2	3	3	3	3	3	3	3	3	3	3	3	3	3	3	3	3	3	3	3	3	3	4	4	4	4	4	4	4	4	4	4	4	4	4	4	4	4	4	4	5	5	5	5	5	5	5	5	5	5	5	5	5	5	5	5	5	5	5	5	5	5	5	5	5	5	5	5	5
					6	6	7	7	8	1	1	2	3	3	4	4	4	5	5	6	6	6	6	7	7	8	8	8	8	9	0	0	1	1	3	4	4	4	5	6	6	8	8	8	8	9	9	9	0	0	0	0	1	1	1	1	1	2	2	2	3	3	3	3	3	4	4	5	5	6	6	6	6	7	7	7	9
					5	8	8	9	1	1	8	0	8	9	3	6	7	5	8	0	4	6	9	1	8	2	4	5	9	4	0	1	0	7	4	4	7	9	9	2	5	1	3	6	8	0	1	8	1	5	6	7	0	3	4	6	7	0	2	3	1	5	6	7	8	4	6	2	7	3	5	7	8	2	4	6	5
					I	P	D	A	Q	D	I	E	T	M	M	A	A	G	V	N	L	L	A	I	E	G	R	M	S	H	M	T	A	N	A	N	Q	S	T	I	F	S	I	P	D	T	A	I	N	K	D	A	L	T	N	V	L	T	P	V	S	P	T	V	T	V	L	Q	K	V	L	D	Y	A	S	S	Y
2X 01	40	13.3	0	0.0	–	–	–	–	–	–	–	–	–	–	–	–	–	–	–	–	–	–	–	–	–	–	–	–	–	–	–	–	–	–	–	–	–	–	–	–	–	–	–	–	–	–	–	–	–	–	–	–	–	–	–	–	–	–	–	–	–	–	–	–	–	–	–	–	–	–	–	–	–	–	–	–	–
2X 02	28	9.3	1	0.3	–	–	–	–	–	–	–	–	–	–	–	–	–	–	–	–	–	–	–	–	–	–	–	–	–	–	–	–	–	–	–	–	–	–	–	–	–	–	–	–	–	–	–	–	–	–	–	–	–	–	–	–	–	–	–	–	–	–	–	–	–	–	–	–	–	–	V	–	–	–	–	–	–
2X 03	2	0.7	1	0.3	–	–	–	S	–	–	–	–	–	–	–	–	–	–	–	–	–	–	–	–	–	–	–	–	–	–	–	–	–	–	–	–	–	–	–	–	–	–	–	–	–	–	–	–	–	–	–	–	–	–	–	–	–	–	–	–	–	–	–	–	–	–	–	–	–	–	–	–	–	–	–	–	–
2X 04	4	1.3	1	0.3	–	–	–	–	–	–	–	–	–	–	–	–	–	–	–	–	–	–	–	–	–	–	–	T	–	–	–	–	–	–	–	–	–	–	–	–	–	–	–	–	–	–	–	–	–	–	–	–	–	–	–	–	–	–	–	–	–	–	–	–	–	–	–	–	–	–	–	–	–	–	–	–	–
2X 05	1	0.3	1	0.3	–	–	–	–	–	–	–	–	–	–	–	–	–	–	–	–	–	–	–	–	–	–	–	–	–	–	–	–	–	–	–	–	–	–	–	–	–	–	–	–	–	–	–	–	–	–	–	–	–	–	–	–	–	K	–	–	–	–	–	–	–	–	–	–	–	–	–	–	–	–	–	–	–
2X 06	1	0.3	1	0.3	–	–	–	–	–	–	–	–	–	–	–	–	–	–	–	–	–	–	–	–	–	–	–	–	–	–	–	–	–	–	–	–	–	–	–	–	–	–	–	–	–	–	–	–	–	–	–	–	–	–	–	–	–	–	–	–	–	–	–	–	–	–	–	–	–	–	–	–	–	D	–	–	–
2X 07	1	0.3	1	0.3	–	–	–	–	–	–	–	–	–	–	–	–	–	–	–	–	–	–	–	–	–	–	–	–	–	–	–	–	–	–	–	–	–	–	–	–	–	–	–	–	–	–	–	–	–	–	–	–	–	–	–	–	–	–	–	–	–	–	–	–	–	–	–	–	R	–	–	–	–	–	–	–	–
2X 08	14	4.7	2	0.6	–	–	–	–	–	–	–	–	–	–	–	–	–	–	–	–	–	–	–	–	–	–	–	–	–	–	–	–	–	–	–	–	–	–	–	–	–	–	–	–	N	–	–	–	–	–	–	–	–	–	–	–	–	–	–	–	–	–	–	–	–	–	–	–	–	–	V	–	–	–	–	–	–
2X 09	1	0.3	2	0.6	–	–	–	–	–	–	–	–	–	–	–	–	–	–	–	–	–	–	–	–	–	–	–	T	–	–	–	–	–	–	–	–	–	–	–	–	–	L	–	–	–	–	–	–	–	–	–	–	–	–	–	–	–	–	–	–	–	–	–	–	–	–	–	–	–	–	–	–	–	–	–	–	–
2X 10	2	0.7	8	2.4	–	–	–	–	–	–	–	–	–	–	–	–	–	–	–	–	–	–	–	–	–	–	–	–	–	–	–	–	–	–	–	–	–	–	–	–	–	–	–	N	–	–	–	–	–	–	–	–	–	–	–	–	–	–	–	–	–	–	–	–	–	–	V	–	–	–	S	N	N	V	A	N	–
2X 11	1	0.3	8	2.4	–	–	–	–	–	–	–	–	–	–	–	–	–	–	–	–	–	–	–	–	–	–	–	–	–	–	–	–	–	–	–	–	–	–	–	–	–	–	–	–	–	–	–	–	–	–	–	–	–	–	–	–	–	–	–	–	–	–	–	N	–	–	V	–	–	–	S	N	N	V	A	N	–
2X 12	3	1.0	8	2.4	–	–	–	–	–	–	–	–	–	–	–	–	–	–	–	–	–	–	–	–	–	–	–	–	–	–	–	–	–	–	–	–	–	–	–	–	–	–	–	–	–	–	–	–	–	–	–	–	–	–	–	I	–	–	–	–	–	–	–	–	–	–	V	–	–	T	E	N	N	V	–	H	–
2X 13	1	0.3	8	2.4	–	–	–	–	–	–	–	–	–	–	–	–	–	–	–	–	–	–	–	–	–	–	–	–	–	–	–	–	–	–	–	–	–	–	–	–	–	–	–	–	–	–	–	V	–	–	–	–	–	–	–	–	–	–	–	–	–	–	–	–	–	–	V	–	–	–	S	N	N	V	A	N	–
2X 14	1	0.3	9	2.7	–	–	–	–	–	–	–	–	–	–	–	–	–	–	–	–	–	–	–	–	–	–	G	–	–	–	–	–	–	–	–	–	–	–	–	–	–	–	–	–	–	–	–	–	–	–	–	–	–	–	–	I	–	–	–	–	–	–	-	–	–	–	V	–	–	T	E	N	N	V	–	H	–
2X 15	7	2.3	10	3.0	–	T	–	–	–	–	–	–	–	–	T	–	–	–	–	–	–	–	–	–	–	–	–	–	–	–	–	–	–	–	–	–	–	–	–	–	–	–	–	–	–	–	–	–	–	–	–	–	–	–	–	–	–	–	A	–	–	–	-	–	–	–	V	–	–	–	S	N	N	V	A	N	–
2X 16	1	0.3	10	3.0	–	T	N	–	–	–	L	K	A	–	T	–	–	–	–	–	–	V	V	–	–	–	G	–	–	–	–	–	–	–	–	–	–	–	–	–	–	–	–	–	–	–	–	–	–	–	–	–	–	–	–	–	–	–	–	–	–	–	–	–	–	–	–	–	–	–	–	N	–	–	–	–	–
2X 17	4	1.3	11	3.3	–	T	N	–	–	–	L	K	A	–	T	–	–	–	–	–	–	–	V	–	–	T	G	–	L	–	–	–	–	–	–	–	–	–	–	–	–	–	–	–	–	–	–	–	–	–	–	–	–	–	–	–	–	–	–	–	–	–	–	–	–	–	–	–	–	–	–	N	–	–	–	–	–
2X 18	1	0.3	14	4.1	–	–	–	–	–	–	–	–	–	–	–	–	–	S	Y	–	–	–	V	–	D	T	–	–	L	L	–	S	T	–	–	–	–	–	–	–	–	–	–	–	–	–	–	–	V	–	–	–	–	–	–	H	–	–	–	–	–	K	–	–	–	–	–	–	–	–	–	N	–	–	–	N	–
2X 19	1	0.3	15	4.4	–	T	N	–	–	–	L	K	–	–	T	–	–	–	–	D	–	–	V	T	–	–	–	–	–	L	–	–	–	–	–	–	–	–	–	–	–	–	–	–	–	–	–	–	–	–	–	–	–	–	H	–	–	–	–	–	–	–	–	–	I	–	–	–	–	–	T	N	H	–	–	N	–
2X 20	1	0.3	16	4.7	–	T	N	–	–	–	L	K	–	–	T	–	–	–	–	–	–	–	–	–	D	T	G	–	L	–	–	S	–	–	S	–	M	A	–	–	–	–	–	–	–	–	–	–	–	–	–	–	–	–	–	I	–	–	–	–	–	–	–	–	–	–	–	–	–	–	–	N	–	–	–	N	–
2X 21	1	0.3	18	5.3	L	–	–	–	L	N	–	–	–	–	T	S	S	S	Y	–	–	–	V	–	D	T	–	–	L	L	–	S	T	–	–	–	–	–	–	–	–	–	–	–	–	–	–	–	–	–	–	–	–	–	–	I	–	–	–	–	–	–	–	–	–	–	–	–	–	–	–	N	–	–	–	N	–
2X 22	4	1.3	18	5.3	–	–	–	–	–	–	–	–	–	–	–	–	–	–	–	–	–	–	–	–	–	–	–	–	–	–	–	–	–	–	–	–	–	–	–	L	–	–	L	–	–	S	V	–	–	–	A	–	S	E	H	–	M	–	–	–	–	–	N	–	N	–	–	V	–	T	E	N	N	V	–	H	–
2X 23	1	0.3	19	5.6	–	–	–	–	–	–	–	–	–	–	–	–	–	–	–	–	–	–	–	–	–	–	–	–	–	–	–	–	–	–	–	–	M	–	–	L	–	–	L	–	–	S	V	–	–	–	A	–	S	E	H	–	M	–	–	–	–	–	N	–	N	–	–	V	–	T	E	N	N	V	–	H	–
2X 24	1	0.3	19	5.6	–	–	–	–	–	–	–	–	–	–	–	–	–	–	–	–	–	–	–	–	–	–	–	–	–	–	–	–	–	–	S	–	M	A	–	–	–	T	L	–	N	–	V	–	–	–	–	–	Q	–	H	–	–	–	–	R	–	A	–	–	N	–	–	V	–	–	S	N	N	V	A	N	–
2X 25	1	0.3	19	5.6	–	–	–	–	–	–	–	–	–	–	–	–	–	S	Y	–	–	–	–	–	D	T	G	–	L	–	–	S	–	–	–	–	M	A	–	L	–	–	–	L	–	–	–	–	–	–	–	–	–	–	–	–	I	–	–	–	–	–	–	–	–	–	–	V	–	–	S	N	N	V	A	N	–
2X 26	1	0.3	25	7.4	–	T	N	–	–	–	L	K	–	–	T	–	–	–	–	–	–	V	–	–	–	–	–	–	L	–	–	–	–	–	–	–	–	–	–	L	–	–	–	T	N	S	V	–	K	E	–	–	–	–	–	I	–	–	–	L	Q	–	I	I	–	–	V	–	–	–	S	N	–	V	T	N	–
2X 27	2	0.7	31	9.2	L	–	–	V	–	–	–	K	A	–	T	–	S	–	Y	–	–	–	–	–	A	S	G	T	–	–	–	–	–	S	–	–	M	–	–	L	–	–	L	–	–	S	V	–	–	–	A	–	S	D	Y	–	M	–	–	–	–	–	N	–	N	–	–	V	–	T	E	N	N	V	–	H	–
2X 28	32	10.7	35	10.4	L	–	–	–	L	N	–	–	A	–	T	S	S	S	Y	–	F	–	–	T	–	T	G	–	L	–	–	S	–	K	–	S	–	–	–	L	–	–	–	T	N	S	V	–	K	E	–	T	–	–	–	I	–	–	–	L	Y	–	I	I	–	–	V	–	–	–	S	N	–	–	T	N	–
2X 29	1	0.3	36	10.7	L	–	–	–	L	N	–	–	A	–	T	S	S	S	Y	–	F	–	–	T	–	T	G	–	L	–	–	S	–	K	–	S	–	–	I	L	–	–	–	T	N	S	V	–	K	E	–	T	–	–	–	I	–	–	–	L	Y	–	I	I	–	–	V	–	–	–	S	N	–	–	T	N	–
2X 30	6	2.0	36	10.7	L	–	–	–	L	N	–	–	A	–	T	S	S	S	Y	–	F	–	–	T	–	T	G	–	L	–	–	S	–	K	–	S	–	–	–	L	–	–	–	T	N	S	V	–	–	–	E	–	T	N	H	I	–	–	–	L	Y	–	I	I	–	–	V	–	–	–	S	N	–	–	T	N	–
2X 31	3	1.0	36	10.7	–	T	–	–	L	N	–	–	A	–	T	S	S	S	Y	–	F	–	–	T	–	T	G	–	L	–	–	S	–	K	–	S	–	–	–	L	–	–	–	T	N	S	V	–	–	–	E	–	T	N	H	I	–	–	–	L	Y	–	I	I	–	–	V	–	–	–	S	N	–	–	T	N	–
2X 32	1	0.3	36	10.7	–	T	–	–	L	N	–	K	A	–	T	–	–	S	Y	–	F	–	–	T	A	T	G	–	L	–	–	S	–	K	–	S	–	–	–	L	–	–	–	T	N	S	V	–	–	–	E	–	T	N	H	I	–	–	–	L	Y	–	I	I	–	–	V	–	–	–	S	N	–	–	T	N	–
2X 33	2	0.7	37	10.9	L	–	–	–	L	N	–	–	A	–	T	S	S	S	Y	–	F	–	–	T	–	T	G	–	L	–	–	S	–	K	–	S	–	–	–	L	–	–	–	T	N	S	V	–	–	–	E	–	T	N	H	I	–	–	–	L	Y	–	I	I	–	–	V	–	–	–	S	N	–	V	T	N	–
2X 34	7	2.3	37	10.9	–	–	–	–	L	N	–	–	A	–	T	S	S	S	Y	–	F	–	–	T	D	T	G	–	L	–	–	S	–	K	–	S	–	–	–	L	–	–	–	T	N	S	V	–	–	–	E	–	T	N	H	I	–	–	–	L	Y	–	I	I	–	–	V	–	–	–	S	N	–	V	T	N	–
2X 35	11	3.7	37	10.9	–	T	N	–	–	–	L	K	A	–	T	S	S	S	Y	–	F	–	–	T	–	T	G	–	L	–	–	S	–	K	–	S	–	–	–	L	–	–	–	T	N	S	V	–	–	–	E	–	T	N	H	I	–	–	–	L	Y	–	I	I	–	–	V	–	–	–	S	N	–	–	T	N	–
2X 36	1	0.3	38	11.2	–	T	N	–	–	N	–	–	A	–	T	S	S	S	Y	–	F	–	–	T	–	T	G	–	L	–	–	S	–	K	–	S	–	–	–	L	–	–	–	T	N	S	V	–	K	E	–	T	T	N	H	I	–	–	–	L	Y	–	I	I	–	–	V	–	–	–	S	N	–	–	T	N	–
2X 37	49	16.3	38	11.2	L	–	–	–	L	N	–	–	A	–	T	S	S	S	Y	–	F	–	–	T	D	T	G	–	L	–	–	S	–	K	–	S	–	–	–	L	–	–	–	T	N	S	V	–	–	–	E	–	T	N	H	I	–	–	–	L	Y	–	I	I	–	–	V	–	–	–	S	N	–	V	T	N	–
2X 38	21	7.0	38	11.2	L	–	–	–	L	N	–	–	A	–	T	S	S	S	Y	–	F	–	–	T	D	T	G	–	L	–	–	S	T	–	–	S	–	–	–	L	–	–	–	T	N	S	V	–	–	–	E	–	T	N	H	I	–	–	–	L	Y	–	I	I	–	–	V	–	–	–	S	N	–	V	T	N	–
2X 39	4	1.3	38	11.2	L	–	–	–	L	N	–	–	A	F	–	S	S	S	Y	–	F	–	–	T	–	T	G	–	–	–	T	S	–	K	–	S	–	–	–	L	–	–	–	T	N	S	V	–	–	–	E	–	T	N	H	I	–	–	–	L	Y	–	I	I	–	–	V	–	–	–	S	N	–	V	T	N	F
2X 40	1	0.3	38	11.2	–	–	–	–	–	N	–	–	A	F	–	S	S	S	Y	–	F	–	–	T	A	T	G	–	L	–	T	S	–	K	–	S	–	–	–	L	–	–	–	T	N	S	V	–	–	–	E	–	T	N	H	I	–	–	–	L	Y	–	I	I	–	–	V	–	–	–	S	N	–	V	T	N	F
2X 41	33	11.0	40	11.8	L	–	–	–	L	N	–	–	A	F	–	S	S	S	Y	–	F	–	–	T	A	T	G	–	L	–	T	S	–	K	–	S	–	–	–	L	–	–	–	T	N	S	V	–	–	–	E	–	T	N	H	I	–	–	–	L	Y	–	I	I	–	–	V	–	–	–	S	N	–	V	T	N	F
2X 42	1	0.3	42	12.4	L	–	–	–	L	N	–	–	A	F	–	S	S	S	Y	–	F	M	V	T	G	T	G	–	L	–	T	S	–	K	–	S	–	–	–	L	–	–	–	T	N	S	V	–	–	–	E	–	T	N	H	I	–	–	–	L	Y	–	I	I	–	–	V	–	–	–	S	N	–	V	T	N	F
2X 43	1	0.3	39	11.5	L	–	–	–	L	N	–	–	A	–	T	S	S	S	Y	–	F	–	–	T	D	T	G	–	L	–	–	S	–	K	–	S	–	–	–	L	–	–	–	T	N	S	V	–	–	–	E	V	T	N	H	I	–	–	–	L	Y	–	I	I	–	–	V	–	–	–	S	N	–	V	T	N	–

### Variations of the *pbp2b* Gene

Compared with the reference strain R6 (GenBank accession No. NC003098), a total of 372 amino acids from positions 305 to 676 of the PBP2b protein were analyzed for the isolates. A total of 111 different substitutions and two insertion variations at 88 amino acid positions were detected in the PBP2b sequence. The two insertion variations were only found in two strains, both located between amino acids 424-425, and the insertion sequences were YIW (Tyr–Ile-Try) and YTW (Tyr-Thr-Try). The most common substitution was E476G (Glu → Gly), accounting for 64.7% (194/300) of the isolates, followed by T446A (Thr → Ala), Q438E (Gln → Glu), L455I (Leu → Ile), which accounted for 63.3% (190/300), 61.7% (185/300) and 61.0% (183/300), respectively. According to the distribution of various substitutions in PBP2b, all strains could be divided into 46 groups. Except for group 2B45 and 2B46, each containing one insertion variation, the other groups all had amino acid substitutions in PBP2b protein. The number of substituted amino acid sites ranged from 0 to 45, accounting for 0-12.1% of the total number of amino acids (*n* = 372) analyzed. Group 2B01 was the most common, accounting for 17.3% of the strains (52/300), and had exactly the same sequence of PBP2b as reference strain R6. The second common group was 2B20, accounting for 14.3% of the strains (43/300). A total of 13 amino acid substitutions were detected in the PBP2b sequence of the strains in this group, and the substitution rate was 3.5% (Table 8). According to the distribution of PEN MICs, the number of substitution sites in strains with MIC ≤ 0.12μg/ml were less than ten, while the number of detectable substitution sites in strains with MIC = 0.25, 0.5, 1, 2, and 4μg/ml were 12, 75, 61, 52, and 54, respectively (Supplementary Table 3).

**TABLE 8:** Deduced amino acid sequences of PBP2b in 300 *S. pneumoniae* isolates.

PBP2b group	n	Percent (%)	Number of substitution sites	Substitution rate (%)	3	3	3	3	3	3	3	3	3	3	3	3	4	4	4	4	4	4	4	4	4	4	4	4	4	4	4	4	4	4	4	4	4	4	4	4	4	4	4	4	4	4	5	5	5	5	5	5	5	5	5	5	5	5	5	5	5	5	5	5	5	5	5	5	5	5	5	5	5	5	5	5	5	6	6	6	6	6	6	6	6	6	6	6	6	6	6	6	
					1	1	2	2	3	5	6	6	6	6	7	8	0	1	1	1	1	2	2	2	2	2	2	3	3	3	3	3	3	4	4	5	6	6	7	7	7	8	8	8	9	9	0	0	0	0	1	1	1	2	3	3	4	4	5	5	5	6	6	6	6	6	6	7	7	7	8	8	8	8	9	9	9	0	0	1	2	2	2	2	4	4	5	6	6	7	7	7	
					5	9	1	2	3	7	1	4	6	9	2	3	6	1	2	4	9	2	4	6	7	8	9	0	1	2	7	8	9	6	9	5	7	9	0	3	6	0	3	9	0	7	2	3	6	7	2	0	6	0	3	8	2	5	2	5	6	1	5	6	7	8	9	1	4	8	1	2	3	5	2	5	7	6	9	9	4	5	8	7	0	1	9	0	4	4	5	6	
					A	S	D	A	E	S	I	D	K	E	P	V	Q	Q	S	V	A	N	Ins	T	Q	A	Y	G	S	F	V	Q	A	T	V	L	G	S	N	S	E	S	G	T	A	D	F	V	E	S	Y	A	A	F	A	N	V	R	G	D	K	D	Q	L	Q	P	T	M	V	D	M	S	I	H	A	T	G	N	L	A	A	D	Q	T	S	D	N	G	S	Q	K	Y	
2B 01	52	17.3	0	0.0	–	–	–	–	–	–	–	–	–	–	–	–	–	–	–	–	–	–	–	–	–	–	–	–	–	–	–	–	–	–	–	–	–	–	–	–	–	–	–	–	–	–	–	–	–	–	–	–	–	–	–	–	–	–	–	–	–	–	–	–	–	–	–	–	–	–	–	–	–	–	–	–	–	–	–	–	–	–	–	–	–	–	–	–	–	–	–	–	
2B 02	3	1.0	1	0.3	–	–	–	–	–	–	–	–	–	–	–	–	–	–	–	–	–	–	–	–	–	–	–	–	–	–	–	–	–	–	–	–	–	–	–	–	–	–	–	–	–	–	–	–	–	–	–	–	–	–	–	–	–	–	–	–	–	–	–	–	–	–	–	I	–	–	–	–	–	–	–	–	–	–	–	–	–	–	–	–	–	–	–	–	–	–	–	–	
2B 03	3	1.0	1	0.3	–	–	–	–	–	–	–	–	–	–	–	–	–	–	–	–	–	–	–	–	–	–	–	–	–	–	–	–	–	–	–	–	–	–	–	–	–	–	–	–	–	–	–	–	–	–	–	–	P	–	–	–	–	–	–	–	–	–	–	–	–	–	–	–	–	–	–	–	–	–	–	–	–	–	–	–	–	–	–	–	–	–	–	–	–	–	–	–	
2B 04	27	9.0	1	0.3	–	–	–	–	–	–	–	–	–	–	–	–	–	–	–	–	–	–	–	–	–	–	–	–	–	–	–	–	–	–	–	–	–	–	–	–	–	–	–	–	–	–	–	–	–	–	–	–	–	–	–	–	–	–	–	–	–	–	–	–	–	–	–	–	–	–	–	–	–	–	–	–	E	–	–	–	–	–	–	–	–	–	–	–	–	–	–	–	
2B 05	1	0.3	1	0.3	–	–	–	–	–	–	–	–	–	–	–	–	–	–	–	–	–	–	–	–	–	–	–	–	–	–	–	–	–	–	–	–	–	–	–	–	–	–	–	–	–	–	–	–	–	–	–	–	–	–	–	–	–	–	–	–	–	–	N	–	–	–	–	–	–	–	–	–	–	–	–	–	–	–	–	–	–	–	–	–	–	–	–	–	–	–	–	–	
2B 06	4	1.3	1	0.3	–	–	–	–	–	–	–	–	–	–	–	–	–	–	–	–	–	–	–	–	–	–	–	–	–	–	–	I	–	–	–	–	–	–	–	–	–	–	–	–	–	–	–	–	–	–	–	–	–	–	–	–	–	–	–	–	–	–	–	–	–	–	–	–	–	–	–	–	–	–	–	–	–	–	–	–	–	–	–	–	–	–	–	–	–	–	–	–	
2B 07	1	0.3	1	0.3	–	–	–	–	–	–	–	–	–	–	–	–	–	–	–	–	–	–	–	–	–	–	–	–	–	–	–	–	–	–	–	–	–	–	–	–	–	–	–	–	–	–	–	–	–	–	–	–	–	–	S	–	–	–	–	–	–	–	–	–	–	–	–	–	–	–	–	–	–	–	–	–	–	–	–	–	–	–	–	–	–	–	–	–	–	–	–	–	
2B 08	2	0.7	1	0.3	T	–	–	–	–	–	–	–	–	–	–	–	–	–	–	–	–	–	–	–	–	–	–	–	–	–	–	–	–	–	–	–	–	–	–	–	–	–	–	–	–	–	–	–	–	–	–	–	–	–	–	–	–	–	–	–	–	–	–	–	–	–	–	–	–	–	–	–	–	–	–	–	–	–	–	–	–	–	–	–	–	–	–	–	–	–	–	–	
2B 09	4	1.3	1	0.3	–	N	–	–	–	–	–	–	–	–	–	–	–	–	–	–	–	–	–	–	–	–	–	–	–	–	–	–	–	–	–	–	–	–	–	–	–	–	–	–	–	–	–	–	–	–	–	–	–	–	–	–	–	–	–	–	–	–	–	–	–	–	–	–	–	–	–	–	–	–	–	–	–	–	–	–	–	–	–	–	–	–	–	–	–	–	–	–	
2B 10	3	1.0	2	0.5	–	–	–	–	–	–	–	–	–	–	–	–	–	–	–	–	–	–	–	–	–	–	–	–	–	–	–	–	–	–	–	–	–	D	–	–	–	–	–	–	–	–	–	–	–	–	–	–	–	–	–	–	–	–	–	–	–	–	–	–	–	–	–	–	–	–	–	–	–	–	–	–	E	–	–	–	–	–	–	–	–	–	–	–	–	–	–	–	
2B 11	4	1.3	2	0.5	–	–	–	–	–	–	–	–	–	–	–	–	–	–	–	–	–	–	–	–	–	–	–	–	–	–	–	–	–	–	–	–	–	–	–	–	–	–	–	–	–	–	–	–	–	–	–	–	–	–	–	D	–	–	–	–	–	–	–	–	–	–	–	–	–	–	–	–	–	–	–	–	E	–	–	–	–	–	–	–	–	–	–	–	–	–	–	–	
2B 12	1	0.3	2	0.5	–	–	–	–	–	–	–	–	–	–	–	–	–	–	–	–	–	–	–	–	–	–	–	–	–	–	–	–	–	–	–	–	–	–	–	–	–	–	–	–	–	–	–	–	–	–	–	–	–	–	–	D	–	–	–	–	–	–	–	–	–	–	–	I	–	–	–	–	–	–	–	–	–	–	–	–	–	–	–	–	–	–	–	–	–	–	–	–	
2B 13	1	0.3	2	0.5	–	–	E	–	–	–	–	–	–	–	–	–	–	–	–	–	–	–	–	–	–	–	–	–	–	–	–	–	–	–	–	–	–	–	–	–	–	–	–	–	–	–	–	–	–	–	–	–	–	–	–	–	–	–	–	–	–	–	–	–	–	–	–	–	–	–	–	–	–	–	–	–	E	–	–	–	–	–	–	–	–	–	–	–	–	–	–	–	
2B 14	1	0.3	6	1.6	–	–	–	–	G	–	L	–	–	–	–	–	–	–	–	–	–	–	–	–	–	–	–	–	–	–	–	–	–	–	–	–	–	–	K	–	G	–	–	S	–	–	–	–	–	–	–	–	–	–	–	D	–	–	–	–	–	–	–	–	–	–	–	–	–	–	–	–	–	–	–	–	–	–	–	–	–	–	–	–	–	–	–	–	–	–	–	–	
2B 15	1	0.3	9	2.4	–	–	–	–	–	–	–	–	–	–	–	–	–	–	–	–	–	–	–	–	–	–	–	–	–	–	–	–	–	A	–	–	–	–	–	–	G	–	–	A	–	–	–	–	D	N	F	–	–	–	–	D	–	–	–	–	–	–	–	–	–	–	–	–	–	–	–	–	V	–	–	–	–	–	–	–	–	–	E	–	–	–	–	–	–	–	–	–	
2B 16	1	0.3	12	3.2	–	–	–	–	–	–	–	–	–	–	–	–	–	–	–	–	–	–	–	–	–	–	–	–	–	–	–	E	–	A	–	I	L	N	–	–	G	–	A	A	S	–	–	I	–	N	–	–	–	–	–	D	–	–	–	–	–	–	–	–	–	–	–	–	–	–	–	–	–	–	–	–	–	–	–	–	–	–	–	–	–	–	–	–	–	–	–	–	
2B 17	7	2.3	12	3.2	–	–	–	–	G	–	L	–	–	–	–	–	–	–	P	–	–	–	–	K	–	–	–	–	–	–	–	E	–	A	–	I	–	–	–	–	G	–	–	S	–	–	–	–	–	–	F	–	–	–	–	D	–	–	–	–	–	–	–	–	–	–	I	–	–	–	–	–	–	–	–	–	–	–	–	–	–	–	–	–	–	–	–	–	–	–	–	–	
2B 18	1	0.3	12	3.2	–	–	–	–	–	–	–	–	–	–	–	–	–	–	P	–	–	Y	–	K	L	–	–	–	–	–	–	E	–	A	–	I	–	–	–	T	G	A	–	A	–	–	–	–	–	–	–	–	–	–	–	D	–	–	–	–	–	–	–	–	–	–	–	–	–	–	–	–	–	–	–	–	–	–	–	–	–	–	–	–	–	–	–	–	–	–	–	–	
2B 19	1	0.3	12	3.2	–	–	–	–	G	–	–	–	–	–	–	–	–	–	P	–	–	Y	–	K	L	–	–	–	–	–	–	E	–	A	–	I	–	–	–	T	G	A	–	A	–	–	–	–	–	–	–	–	–	–	–	–	–	–	–	–	–	–	–	–	–	–	–	–	–	–	–	–	–	–	–	–	–	–	–	–	–	–	–	–	–	–	–	–	–	–	–	–	
2B 20	43	14.3	13	3.5	–	–	–	–	G	–	–	–	–	–	–	–	–	–	P	–	–	Y	–	K	L	–	–	–	–	–	–	E	–	A	–	I	–	–	–	T	G	A	–	A	–	–	–	–	–	–	–	–	S	–	–	–	–	–	–	–	–	–	–	–	–	–	–	–	–	–	–	–	–	–	–	–	–	–	–	–	–	–	–	–	–	–	–	–	–	–	–	–	
2B 21	1	0.3	13	3.5	–	–	–	–	–	–	–	–	–	–	–	–	–	–	P	–	–	Y	–	K	L	–	–	–	–	–	–	E	T	A	–	I	–	–	–	T	G	A	–	A	–	–	–	–	–	–	–	–	–	–	–	D	–	–	–	–	–	–	–	–	–	–	–	–	–	–	–	–	–	–	–	–	–	–	–	–	–	–	–	–	–	–	–	–	–	–	–	–	
2B 22	6	2.0	13	3.5	–	–	–	–	–	–	–	–	–	–	–	–	–	–	–	–	–	–	–	–	–	–	–	–	–	–	–	E	–	A	–	I	L	N	–	–	G	–	A	A	S	–	–	I	–	N	–	–	–	–	–	D	–	–	–	–	–	–	–	–	–	–	–	–	–	–	–	–	V	–	–	–	–	–	–	–	–	–	–	–	–	–	–	–	–	–	–	–	
2B 23	1	0.3	13	3.5	–	–	–	–	G	–	–	–	–	–	–	–	–	–	–	–	–	–	–	–	A	F	S	R	P	M	–	–	–	A	–	–	–	–	–	–	G	–	–	S	–	–	–	I	–	–	–	–	–	–	–	D	–	–	–	–	–	–	–	–	–	–	–	–	–	–	–	–	V	–	–	–	–	–	–	–	–	–	–	–	–	–	–	–	–	–	–	–	
2B 24	10	3.3	14	3.8	–	–	–	–	G	–	L	–	–	–	–	–	–	–	P	–	–	Y	–	K	L	–	–	–	–	–	–	E	–	A	–	I	–	–	–	T	G	A	–	A	–	–	–	–	–	–	–	–	–	–	–	D	–	–	–	–	–	–	-	–	–	–	–	–	–	–	–	–	–	–	–	–	–	–	–	–	–	–	–	–	–	–	–	–	–	–	–	–	
2B 25	1	0.3	14	3.8	–	–	–	–	G	–	–	–	–	–	–	–	P	–	P	–	–	Y	–	K	L	–	–	–	–	–	–	E	–	A	–	I	–	–	–	T	G	A	–	A	–	–	–	–	–	–	–	–	S	–	–	–	–	–	–	–	–	–	-	–	–	–	–	–	–	–	–	–	–	–	–	–	–	–	–	–	–	–	–	–	–	–	–	–	–	–	–	–	
2B 26	22	7.3	15	4.0	–	–	–	–	G	–	–	–	N	–	Q	–	–	–	P	–	–	Y	–	K	L	–	–	–	–	–	–	E	–	A	–	I	–	–	–	T	G	A	–	A	–	–	–	–	–	–	–	–	–	–	–	D	–	–	–	–	–	–	-	–	–	–	–	–	–	–	–	–	–	–	–	–	–	–	–	–	–	–	–	–	–	–	–	–	–	–	–	–	
2B 27	4	1.3	15	4.0	–	–	–	–	G	–	–	–	–	–	–	–	–	–	P	–	–	Y	–	K	L	–	–	–	–	–	–	E	–	A	–	I	–	–	–	T	G	A	–	A	–	–	–	–	D	–	–	–	–	–	–	D	–	–	–	–	–	–	–	–	–	–	–	–	–	–	–	–	–	–	–	–	–	–	–	–	–	–	E	–	–	–	–	–	–	–	–	–	
2B 28	2	0.7	22	5.9	–	–	–	–	G	–	L	–	N	–	Q	–	–	–	P	–	–	Y	–	K	L	–	–	–	–	–	–	E	–	A	–	–	–	–	–	–	G	–	–	S	–	–	–	–	D	–	–	–	–	–	–	D	–	–	–	–	–	–	–	–	–	–	–	–	–	–	–	–	–	–	–	–	–	–	A	–	E	G	E	N	–	–	–	–	–	N	Q	H	
2B 29	2	0.7	45	12.1	–	–	–	–	G	–	L	–	N	–	Q	–	–	–	P	–	–	Y	–	K	L	–	–	–	–	–	–	E	–	A	–	I	–	–	–	T	G	A	–	A	–	–	–	–	D	–	–	–	–	–	–	D	L	H	D	–	–	E	A	I	D	T	K	I	–	E	–	A	–	–	S	–	P	D	T	G	–	G	E	N	T	E	K	N	A	N	Q	H	
2B 30	1	0.3	17	4.6	–	–	–	–	G	–	–	–	–	–	–	–	–	–	P	–	–	Y	–	K	L	–	–	–	–	–	–	E	–	A	–	I	–	–	–	T	G	A	–	A	–	–	–	–	D	–	–	–	–	–	–	–	–	–	–	–	–	–	–	–	–	–	–	–	–	–	–	–	–	–	–	–	–	–	A	–	–	G	E	N	–	–	–	–	–	–	–	–	
2B 31	2	0.7	17	4.6	–	–	–	–	–	–	–	–	–	–	–	–	–	–	P	–	–	Y	–	K	L	–	–	–	–	–	–	E	–	A	–	I	–	–	–	T	G	A	–	A	–	–	–	I	–	N	F	–	–	–	–	D	–	–	–	–	–	–	–	–	–	–	–	–	–	–	–	–	V	–	–	–	–	–	–	–	–	–	E	–	–	–	–	–	–	–	–	–	
2B 32	1	0.3	17	4.6	–	–	–	–	–	–	–	–	–	–	–	–	–	–	–	–	–	–	–	–	A	F	S	V	P	–	–	–	–	A	–	–	–	–	–	–	G	–	–	S	–	–	–	I	–	–	–	–	–	–	–	D	–	–	–	–	–	–	–	–	–	–	–	–	–	–	–	–	–	–	–	–	–	–	–	–	–	G	E	N	–	–	–	–	–	N	Q	H	
2B 33	2	0.7	18	4.8	–	–	–	–	–	–	–	–	–	–	–	–	–	–	–	–	–	–	–	–	A	F	S	V	P	–	–	–	–	A	–	–	–	–	–	–	G	–	–	S	–	–	–	I	–	N	F	–	–	–	–	D	–	–	–	–	–	–	– -	–	–	–	–	–	–	–	–	–	V	–	–	–	–	–	A	–	–	G	E	N	–	–	–	–	–	–	–	–	
2B 34	2	0.7	18	4.8	–	–	–	–	G	–	L	–	–	–	–	–	–	–	P	–	–	Y	–	K	L	–	–	–	–	–	–	E	–	A	–	I	–	–	–	T	G	A	–	A	–	–	–	–	–	–	–	–	–	–	–	D	–	–	–	–	–	–	–	–	–	–	–	–	–	–	–	–	–	–	–	–	–	–	–	A	–	–	G	E	N	–	–	–	–	–	–	–	–
2B 35	5	1.7	18	4.8	S	–	–	S	T	A	–	N	E	D	–	–	–	–	P	–	–	Y	–	K	L	–	–	–	–	–	–	E	–	A	–	I	–	–	–	T	G	A	–	A	–	–	–	–	–	–	–	–	–	–	–	–	–	–	–	–	–	–	–	-	–	–	–	–	–	–	–	–	–	–	–	–	–	–	–	–	–	–	–	–	–	–	–	–	–	–	–	–	
2B 36	2	0.7	18	4.8	–	–	–	–	–	–	L	–	–	–	–	–	–	–	P	–	–	Y	–	K	L	–	–	–	–	–	–	E	–	A	–	I	–	–	–	T	G	A	–	A	–	–	–	–	–	–	–	–	S	–	–	D	–	–	–	–	–	–	–	–	–	–	–	–	–	–	–	–	–	–	–	–	–	–	A	–	–	G	E	N	–	–	–	–	–	–	–	–	
2B 37	1	0.3	38	10.2	–	–	–	–	–	–	–	–	–	–	–	–	–	–	P	–	–	–	–	–	–	–	–	–	–	–	–	E	–	A	–	I	L	N	–	–	G	–	A	A	S	–	–	I	–	N	–	–	–	–	G	–	I	H	–	E	Q	N	S	V	E	S	K	–	I	E	V	–	–	Q	–	G	A	–	S	–	E	G	E	N	–	–	–	–	–	N	Q	H	
2B 38	1	0.3	19	5.1	–	–	–	–	–	–	L	–	–	–	–	–	–	H	P	–	–	Y	–	K	L	–	–	–	–	–	–	E	–	A	–	I	–	–	–	T	G	A	–	A	–	–	–	–	–	–	–	–	S	–	–	–	–	D	–	–	–	–	–	– - -	–	–	–	–	–	–	–	–	–	–	–	–	–	–	A	–	–	G	E	N	–	–	–	–	–	–	–	–	
2B 39	1	0.3	32	8.6	–	–	–	–	G	–	L	–	–	D	–	–	–	–	P	–	–	Y	–	K	L	–	–	–	–	–	–	E	–	A	–	I	–	–	–	T	G	A	–	A	–	–	–	–	–	–	–	–	–	–	–	D	–	–	–	–	–	E	A	I	D	T	K	I	–	E	–	A	–	–	–	–	–	–	S	–	E	G	E	N	–	–	–	–	–	N	Q	H	
2B 40	1	0.3	32	8.6	–	–	–	–	G	–	L	–	–	–	–	I	–	–	P	–	–	Y	–	K	L	–	–	–	–	–	–	E	–	A	–	I	–	–	–	T	G	A	–	A	–	Y	–	–	–	–	–	–	–	–	–	D	–	–	–	–	–	–	–	–	–	–	–	–	–	–	–	–	–	–	–	–	P	D	T	G	–	G	E	N	T	E	K	N	A	N	Q	H	
2B 41	1	0.3	37	9.9	–	–	–	–	G	–	L	–	–	–	–	–	–	–	T	F	–	–	–	K	–	–	–	–	–	–	–	–	–	S	I	–	–	–	–	–	G	–	–	S	–	–	L	–	–	–	–	–	T	–	–	D	–	–	–	–	–	E	A	I	D	T	K	I	–	E	–	A	–	–	–	–	P	D	T	G	–	G	D	S	T	E	K	N	A	N	Q	H	
2B 42	1	0.3	38	10.2	–	–	–	–	G	–	–	–	–	–	–	–	–	–	P	–	–	Y	–	K	L	–	–	–	–	–	–	E	–	A	–	I	–	–	–	T	G	A	–	A	–	–	–	–	–	–	–	–	–	–	–	D	–	–	–	–	–	E	A	I	D	T	K	I	–	E	–	A	–	–	–	–	P	D	T	G	–	G	E	N	T	E	K	N	A	N	Q	H	
2B 43	27	9.0	40	10.8	–	–	–	–	G	–	L	–	–	D	–	–	–	–	P	–	–	Y	–	K	L	–	–	–	–	–	–	E	–	A	–	I	–	–	–	T	G	A	–	A	–	–	–	–	–	–	–	–	–	–	–	D	–	–	–	–	–	E	A	I	D	T	K	I	–	E	–	A	–	–	–	–	P	D	T	G	–	G	E	D	T	E	K	N	A	N	Q	H	
2B 44	40	13.3	40	10.8	–	–	–	–	G	–	L	–	N	–	–	–	–	–	P	–	–	Y	–	K	L	–	–	–	–	–	–	E	–	A	–	I	–	–	–	T	G	A	–	A	–	–	–	–	–	–	–	–	–	–	–	D	–	–	–	–	–	E	A	I	D	T	K	I	–	E	–	A	–	–	–	–	P	D	T	G	–	G	E	N	T	E	K	N	A	N	Q	H	
2B 45	1	0.3	9	2.4	–	–	–	–	–	–	L	–	–	–	–	–	–	–	–	–	S	–	YTW	–	–	–	–	–	–	–	–	–	–	S	–	–	–	–	–	–	G	–	–	S	–	–	–	–	–	–	–	–	–	–	–	D	–	–	–	–	–	–	–	–	–	–	–	–	–	–	–	–	V	–	–	–	–	–	–	–	–	–	E	–	–	–	–	–	–	–	–	–	
2B 46	1	0.3	8	2.2	–	–	–	–	G	–	L	–	–	–	–	–	–	–	–	–	S	–	YIW	–	–	–	–	–	–	–	–	–	–	S	–	–	–	–	–	–	G	–	–	S	–	–	–	–	–	–	–	–	–	–	–	D	–	–	–	–	–	–	-	–	–	–	–	–	–	–	–	–	–	–	–	–	–	–	–	–	–	–	–	–	–	–	–	–	–	–	–	–	

### Variations of the *pbp1a* Gene

Compared with the reference strain R6 (GenBank accession No. NC003098), a total of 344 amino acids from positions 312 to 655 of the PBP1a protein were analyzed for the studied isolates. A total of 105 different substitutions at 85 amino acid positions were detected in PBP1a. The E388D substitutions (Glu → Asp) was detected in all the 300 isolates. Apart from this, the most common substitution was S540T (Ser → Thr), accounting for 66.7% (200/300), followed by N546G (Asn → Gly), A550P (Gly → Pro), T574N (Thr → Asn), S575T (Ser → Thr), Q576G (Gln → Gly), F577Y (Phe → Tyr), N609D (Asn → Asp), each being detected in 60.0% (180/300) of the strains. According to the distribution of various substitutions in PBP1a, all strains could be divided into 25 groups. The number of substituted amino acids in each group ranged from 1 to 55, accounting for 0.3 −16.0% of the total number of amino acids analyzed. Based on the above substitution classification, group 1A15 was the most common, accounting for 28.7% (86/300) of the strains. All strains in this group had 43 amino acid substitution sites, with the substitution rate of 12.5% of the total number of amino acids analyzed, followed by group 1A04, accounting for 14.7% (44/300) of all the strains (Table 9). Based on the distribution of PEN MIC, the number of substitution sites in strains with MIC ≤ 0.12 μg/ml were less than ten. The number of substitution sites in strains with MIC = 0.25, 0.5, 1, 2, and 4 μg/ml were 17, 97, 88, 71, and 68, respectively (Supplementary Table 4).

**TABLE 9:** Deduced amino acid sequences of PBP1a in 300 *S. pneumoniae* isolates.

PBP1a group	n	Percent (%)	Number of substitution sites	Substitution rate (%)	3	3	3	3	3	3	3	3	3	3	3	3	3	3	3	3	3	3	3	3	3	4	4	4	4	4	4	4	4	4	4	4	4	4	4	4	5	5	5	5	5	5	5	5	5	5	5	5	5	5	5	5	5	5	5	5	5	5	5	5	5	5	6	6	6	6	6	6	6	6	6	6	6	6	6	6	6	6	6	6	6	6	6	6	6
					1	1	1	1	2	2	2	3	3	4	5	5	7	8	8	8	8	9	9	9	9	0	0	0	1	1	2	3	4	5	6	7	7	7	9	9	0	0	1	1	1	1	1	1	2	3	4	4	4	5	5	6	6	7	7	7	7	7	7	8	8	9	0	0	0	1	1	1	1	2	2	2	2	3	3	3	3	3	4	4	4	4	4	5	5
					2	6	7	8	0	1	6	3	7	7	1	8	1	2	5	6	8	2	3	5	7	5	6	8	3	4	1	2	3	9	2	3	4	5	5	7	3	5	2	4	5	6	7	8	0	3	0	3	6	0	3	6	8	0	1	4	5	6	7	3	5	2	6	7	9	1	2	5	6	2	3	8	9	0	2	5	6	8	0	1	3	5	9	0	4
					D	T	D	E	V	A	E	I	S	A	S	I	T	L	G	V	E	T	I	H	E	N	T	V	R	G	L	P	N	I	S	D	K	K	T	Y	H	V	E	E	F	S	N	V	T	D	S	T	N	A	P	E	I	N	H	T	S	Q	F	L	A	S	L	V	N	L	T	A	K	M	T	G	S	N	E	N	I	E	L	Y	N	E	K	N	S
1A 01	40	13.3	1	0.3	–	–	–	–	–	–	–	–	–	–	–	–	–	–	–	–	D	–	–	–	–	–	–	–	–	–	–	–	–	–	–	–	–	–	–	–	–	–	–	–	–	–	–	–	–	–	–	–	–	–	–	–	–	–	–	–	–	–	–	-	–	–	–	–	–	–	–	–	–	–	–	–	–	–	–	–	–	–	–	–	–	–	–	–	–
1A 02	5	1.7	2	0.6	–	–	–	–	–	–	–	–	–	–	–	–	–	–	–	–	D	–	–	–	–	–	–	–	–	–	–	–	–	–	–	–	–	–	–	–	–	–	–	–	–	–	–	–	–	–	–	–	–	–	–	–	–	–	–	–	–	–	–	–	–	–	–	–	–	–	–	–	–	–	–	–	R	–	–	–	–	–	–	–	–	–	–	–	–
1A 03	5	1.7	2	0.6	–	–	–	–	–	–	–	–	–	–	–	–	–	–	–	–	D	–	–	–	–	–	–	–	–	–	–	–	–	–	–	–	–	–	–	–	–	–	–	–	Q	–	–	–	–	–	–	–	–	–	–	–	–	–	–	–	–	–	–	-	–	–	–	–	–	–	–	–	–	–	–	–	–	–	–	–	–	–	–	–	–	–	–	–	–
1A 04	44	14.7	2	0.6	–	–	–	–	–	–	–	–	–	–	–	–	–	–	–	–	D	–	–	–	–	–	–	–	–	–	–	–	–	–	–	–	–	–	–	–	–	–	–	–	–	–	–	–	–	–	–	T	–	–	–	–	–	–	–	–	–	–	–	-	–	–	–	–	–	–	–	–	–	–	–	–	–	–	–	–	–	–	–	–	–	–	–	–	–
1A 05	11	3.7	2	0.6	–	–	–	–	–	–	–	–	–	–	–	–	–	–	–	I	D	–	–	–	–	–	–	–	–	–	–	–	–	–	–	–	–	–	–	–	–	–	–	–	–	–	–	–	–	–	–	–	–	–	–	–	–	–	–	–	–	–	–	-	–	–	–	–	–	–	–	–	–	–	–	–	–	–	–	–	–	–	–	–	–	–	–	–	–
1A 06	8	2.7	2	0.6	–	–	–	–	–	–	–	–	–	–	–	–	–	–	–	–	D	–	–	–	–	–	–	–	–	–	–	–	–	–	–	–	–	–	–	–	–	–	–	–	–	–	–	–	–	–	E	–	–	–	–	–	–	–	–	–	–	–	–	-	–	–	–	–	–	–	–	–	–	–	–	–	–	–	–	–	–	–	–	–	–	–	–	–	–
1A 07	6	2.0	3	0.9	–	–	–	–	–	–	–	–	–	–	–	–	–	–	–	I	D	–	–	–	–	–	–	–	–	–	–	–	–	–	–	–	–	–	–	–	–	–	–	–	–	–	–	–	–	–	–	T	–	–	–	–	–	–	–	–	–	–	–	-	–	–	–	–	–	–	–	–	–	–	–	–	–	–	–	–	–	–	–	–	–	–	–	–	–
1A 08	1	0.3	10	2.9	–	–	–	–	–	–	–	–	–	–	–	–	–	–	–	–	D	–	–	–	–	D	–	–	–	–	–	–	–	L	–	–	–	–	–	–	–	–	–	–	L	–	–	–	–	–	–	T	–	–	–	–	–	–	–	–	–	–	–	–	–	–	–	–	–	–	–	–	–	–	–	–	–	–	–	–	–	D	I	–	–	Q	Q	–	P
1A 09	1	0.3	15	4.4	–	–	–	–	–	–	–	–	–	–	–	–	–	–	–	–	D	–	–	–	–	–	–	–	–	–	–	–	–	–	–	–	–	–	–	–	–	–	–	–	–	–	–	–	–	–	V	I	G	P	–	–	–	K	Y	N	T	G	Y	M	V	A	–	–	D	–	–	–	–	–	–	–	–	–	–	–	–	–	–	–	–	–	–	–	–
1A 10	1	0.3	18	5.2	–	–	–	–	–	–	–	–	–	–	–	–	–	–	–	–	D	–	–	–	–	D	–	–	–	–	–	–	–	L	–	–	–	–	–	–	–	–	–	–	–	–	–	–	–	–	V	I	G	P	–	–	–	K	Y	N	T	G	Y	M	V	A	–	–	D	–	–	–	–	–	–	–	–	–	–	–	V	–	–	–	–	–	–	–	–
1A 11	2	0.7	25	7.3	N	–	–	–	–	–	–	–	T	–	–	–	–	–	–	–	D	–	–	–	–	D	I	–	–	–	–	–	–	–	–	N	–	–	I	H	N	I	–	–	–	–	D	A	–	E	V	I	G	P	–	–	–	K	Y	N	T	G	Y	–	–	–	–	–	D	–	–	–	–	–	–	–	–	–	–	–	V	–	–	–	–	–	–	–	–
1A 12	1	0.3	27	7.8	N	–	–	–	–	–	–	–	T	–	–	–	S	–	–	–	D	–	–	–	–	D	I	–	–	–	–	–	–	L	–	–	Q	–	–	–	–	–	–	–	–	–	–	–	–	–	T	–	G	P	–	–	–	K	Y	N	T	G	Y	M	V	–	I	I	D	F	L	–	–	–	–	–	–	–	–	–	V	D	I	–	–	–	–	–	–
1A 13	1	0.3	36	10.5	–	S	–	–	–	S	D	V	–	–	A	T	A	I	–	–	D	–	M	N	I	S	–	–	–	A	–	T	D	M	A	N	–	Q	–	–	–	–	K	–	–	–	–	–	–	–	T	–	G	P	–	–	V	–	–	N	T	G	Y	M	V	–	I	–	D	F	L	–	–	–	–	–	–	–	–	–	–	–	–	–	–	–	–	–	–
1A 14	1	0.3	37	10.8	–	S	–	–	–	S	D	V	–	–	A	T	S	–	–	–	D	S	–	–	V	D	–	L	H	V	I	T	D	M	A	N	–	Q	–	–	–	–	K	–	–	–	–	–	–	–	T	–	G	P	–	–	V	–	–	N	T	G	Y	M	V	–	I	–	D	F	L	–	–	–	–	–	–	–	–	–	–	–	–	–	–	–	–	–	–
1A 15	86	28.7	43	12.5	–	S	–	–	–	S	D	V	–	–	A	T	S	–	–	–	D	S	–	–	V	D	–	L	H	V	I	T	D	M	A	N	–	–	I	H	N	I	–	–	–	–	D	A	–	E	T	–	G	P	–	–	–	K	Y	N	T	G	Y	M	V	–	I	–	D	F	L	–	–	–	–	–	–	–	–	–	–	–	–	–	–	–	–	–	–
1A 16	9	3.0	43	12.5	–	S	–	–	–	S	D	V	–	–	A	T	S	–	–	–	D	–	M	N	I	S	–	–	–	A	–	T	D	M	A	–	–	–	–	–	–	–	–	–	–	–	–	–	–	–	A	–	G	P	–	D	–	K	–	N	T	G	Y	M	V	–	I	–	D	F	L	–	–	I	S	D	D	Q	G	T	M	D	–	F	–	–	–	–	–
1A 17	3	1.0	44	12.8	–	S	–	–	–	S	D	V	–	–	A	T	S	–	–	–	D	S	–	–	V	D	–	L	H	V	I	T	D	M	A	N	–	–	I	H	N	I	–	–	–	–	D	A	I	E	T	–	G	P	–	–	–	K	Y	N	T	G	Y	M	V	–	I	–	D	F	L	–	–	–	–	–	–	–	–	–	–	–	–	–	–	–	–	–	–
1A 18	35	11.7	45	13.1	–	S	–	–	–	S	D	V	–	–	A	T	A	I	–	–	D	–	M	N	I	S	–	–	–	A	–	T	D	M	A	N	–	Q	–	–	–	–	K	–	–	–	–	–	–	–	T	–	G	P	–	–	V	–	–	N	T	G	Y	M	V	–	I	–	D	F	L	–	–	I	–	D	T	H	–	T	M	D	–	F	–	–	–	–	P
1A 19	9	3.0	46	13.4	–	–	–	–	–	–	–	V	–	–	A	T	A	I	–	–	D	–	M	N	I	S	–	–	–	A	–	T	D	M	A	N	–	Q	I	H	N	–	–	–	–	–	D	–	–	E	A	–	G	P	A	D	–	K	–	N	T	G	Y	M	V	–	I	–	D	F	L	–	–	I	–	D	T	H	–	T	M	D	–	F	–	–	–	–	–
1A 20	2	0.7	49	14.2	–	–	–	–	–	–	–	–	–	S	–	–	A	–	D	I	D	S	–	–	V	D	–	L	K	V	I	T	D	M	A	N	–	–	I	H	N	I	S	–	Y	A	D	P	–	E	A	–	G	P	–	D	–	–	Y	N	T	G	Y	M	V	–	I	–	D	F	Y	–	–	–	–	D	N	–	G	T	M	–	–	–	S	–	–	K	–
1A 21	1	0.3	50	14.5	–	–	–	–	I	–	–	–	–	S	–	–	A	–	D	I	D	S	–	–	V	D	–	L	K	V	I	T	D	M	A	N	–	–	I	H	N	I	S	–	Y	A	D	P	–	E	A	–	G	P	–	D	–	–	Y	N	T	G	Y	M	V	–	I	–	D	F	Y	–	–	–	–	D	N	–	G	T	M	–	–	–	S	–	–	K	–
1A 22	5	1.7	51	14.8	–	S	–	–	–	S	D	V	–	–	A	T	A	I	–	–	D	–	M	N	I	S	–	–	–	A	–	T	D	M	A	N	–	Q	I	H	N	–	K	–	–	–	D	–	–	E	A	–	G	P	A	D	–	K	–	N	T	G	Y	M	V	–	I	–	D	F	L	–	–	I	–	D	T	H	–	T	M	D	–	F	–	–	–	–	–
1A 23	1	0.3	52	15.1	–	S	E	–	–	S	D	V	–	–	A	T	–	I	–	–	D	–	M	N	I	S	–	–	–	A	–	T	D	–	A	N	–	H	I	H	N	I	–	–	–	–	D	A	–	E	A	–	G	P	–	D	–	K	–	N	T	G	Y	M	V	–	I	–	D	F	L	–	–	I	S	D	D	Q	G	T	M	D	–	F	–	–	–	–	–
1A 24	21	7.0	53	15.4	–	S	–	–	–	S	D	V	–	–	A	T	S	–	–	–	D	S	–	–	V	D	–	L	H	V	I	T	D	M	A	N	–	–	I	H	N	I	–	–	–	–	D	A	–	E	T	–	G	P	–	–	–	K	Y	N	T	G	Y	M	V	–	I	I	D	F	L	G	R	–	–	D	N	H	G	T	M	–	–	–	S	–	–	–	–
1A 25	1	0.3	55	16.0	–	S	–	–	–	S	D	V	–	–	A	T	S	–	–	–	D	S	–	–	V	D	–	L	H	V	I	T	D	M	A	N	–	–	I	H	N	I	–	–	–	–	D	A	–	E	T	–	G	P	–	–	–	K	Y	N	T	G	Y	M	V	–	I	I	D	F	L	G	R	–	–	D	N	H	G	T	M	–	–	–	S	–	Q	–	N

### Association Between Serotypes, Penicillin Susceptibility and Penicillin-Binding Proteins Substitution Patterns

Based on a comprehensive analysis of the substitutions of PBP2x, PBP2b and PBP1a, the 300 *S. pneumoniae* isolates could be divided into 101 PBPs substitution combinations: PBP001- PBP101 (Table 10).

**TABLE 10:** Association of serotypes, penicillin susceptibility and substitution patterns of variations in PBP2x, PBP2b and PBP1a in 300 *S. pneumoniae* isolates.

Serotype	MIC (μg/ml)	No.	Substitution patterns of PBPs	Group of PBP2x	Group of PBP2b	Group of PBP1a
1	≤ 0.015	5	P031	2X02	2B 04	1A 04
2	≤ 0.015	1	P022	2X01	2B 01	1A 03
3	≤ 0.015	9	P001	2X01	2B 01	1A 01
	≤ 0.015	1	P003	2X04	2B 01	1A 01
	≤ 0.015	3	P011	2X01	2B 06	1A 01
	≤ 0.015	1	P012	2X05	2B 06	1A 01
	≤ 0.015	1	P013	2X01	2B 07	1A 01
	≤ 0.015	4	P014	2X01	2B 09	1A 01
	≤ 0.015	2	P025	2X04	2B 01	1A 04
	≤ 0.015	1	P026	2X09	2B 01	1A 04
	≤ 0.015	2	P028	2X22	2B 01	1A 04
	≤ 0.015	1	P042	2X02	2B 04	1A 05
	≤ 0.015	1	P043	2X06	2B 04	1A 05
	≤ 0.015	1	P046	2X02	2B 13	1A 05
	≤ 0.015-0.03	4	P047	2X01	2B 01	1A 06
4	≤ 0.015	1	P024	2X01	2B 01	1A 04
5	≤ 0.015	1	P001	2X01	2B 01	1A 01
	≤ 0.015	1	P003	2X04	2B 01	1A 01
6A	0.5	1	P007	2X37	2B 02	1A 01
	0.25	1	P054	2X20	2B 15	1A 09
	2	8	P090	2X37	2B 20	1A 19
	1	1	P091	2X37	2B 42	1A 19
	0.5	1	P099	2X33	2B 39	1A 23
6B	0.25	1	P005	2X15	2B 01	1A 01
	0.5	1	P019	2X16	2B 32	1A 01
	0.25	1	P027	2X15	2B 01	1A 04
	0.5	1	P053	2X18	2B 14	1A 08
	0.5	2	P066	2X37	2B 31	1A 15
	0.5	1	P077	2X34	2B 45	1A 15
	0.5	1	P095	2X41	2B 30	1A 22
	1	2	P097	2X31	2B 36	1A 22
	0.5	1	P098	2X31	2B 38	1A 22
6C	0.25	1	P004	2X10	2B 01	1A 01
	≤ 0.015	1	P036	2X02	2B 11	1A 04
7C	0.03	1	P010	2X22	2B 04	1A 01
	2	1	P096	2X30	2B 34	1A 22
7F	≤ 0.015	2	P015	2X01	2B 11	1A 01
8	≤ 0.015	4	P023	2X02	2B 01	1A 03
9A	≤ 0.015	1	P041	2X08	2B 01	1A 05
	≤ 0.015	1	P044	2X08	2B 04	1A 05
9N	≤ 0.015	1	P027	2X15	2B 01	1A 04
	≤ 0.015	1	P042	2X02	2B 04	1A 05
9V	≤ 0.015	2	P041	2X08	2B 01	1A 05
	≤ 0.015	1	P045	2X11	2B 04	1A 05
10A	0.06	1	P027	2X15	2B 01	1A 04
	≤ 0.015	1	P049	2X01	2B 01	1A 07
	≤ 0.015	1	P051	2X01	2B 05	1A 07
11A	≤ 0.015	1	P006	2X02	2B 02	1A 01
	≤ 0.015	3	P052	2X02	2B 10	1A 07
12F	≤ 0.015	4	P020	2X08	2B 04	1A 02
13	≤ 0.015	1	P029	2X23	2B 01	1A 04
	0.12	1	P033	2X14	2B 04	1A 04
	0.5	1	P056	2X27	2B 33	1A 11
14	0.5	1	P056	2X27	2B 33	1A 11
	0.5	1	P057	2X21	2B 24	1A 12
	1	1	P058	2X37	2B 20	1A 13
	1	1	P065	2X30	2B 28	1A 15
	1	1	P083	2X37	2B 19	1A 18
	0.5	1	P089	2X30	2B 28	1A 18
	2-4	21	P100	2X38	2B 26	1A 24
15A	≤ 0.015	2	P032	2X08	2B 04	1A 04
	0.5	1	P039	2X24	2B 22	1A 04
15B	1	1	P048	2X35	2B 29	1A 06
	1	2	P078	2X35	2B 17	1A 16
	1	1	P079	2X35	2B 29	1A 16
15C	1	5	P078	2X35	2B 17	1A 16
	1	1	P080	2X35	2B 34	1A 16
15F	0.25	1	P036	2X02	2B 11	1A 04
	≤ 0.015	1	P047	2X01	2B 01	1A 06
17	≤ 0.015	1	P047	2X01	2B 01	1A 06
17A	≤ 0.015	1	P031	2X02	2B 04	1A 04
18C	≤ 0.015	1	P042	2X02	2B 04	1A 05
19A	≤ 0.015	1	P024	2X01	2B 01	1A 04
	2	1	P071	2X01	2B 44	1A 15
	2-4	32	P072	2X28	2B 44	1A 15
	2	1	P073	2X29	2B 44	1A 15
	2	1	P074	2X35	2B 44	1A 15
	2	4	P075	2X39	2B 44	1A 15
	2	1	P076	2X41	2B 44	1A 15
19F	0.5	1	P016	2X02	2B 22	1A 01
	0.5	1	P060	2X37	2B 18	1A 15
	0.5	1	P061	2X37	2B 21	1A 15
	1	1	P062	2X30	2B 24	1A 15
	1	1	P063	2X32	2B 24	1A 15
	1	6	P064	2X37	2B 24	1A 15
	4	1	P068	2X36	2B 40	1A 15
	4	1	P069	2X40	2B 41	1A 15
	2-4	27	P070	2X41	2B 43	1A 15
	1	1	P086	2X41	2B 20	1A 18
20	0.5	4	P018	2X17	2B 27	1A 01
	≤ 0.015	3	P032	2X08	2B 04	1A 04
22F	0.5	1	P037	2X22	2B 16	1A 04
23A	0.5	1	P055	2X19	2B 37	1A 10
	0.5	1	P092	2X25	2B 23	1A 20
	1	1	P093	2X30	2B 26	1A 20
	1	1	P094	2X30	2B 24	1A 21
23F	0.5	3	P038	2X12	2B 22	1A 04
	≤ 0.015	1	P040	2X01	2B 01	1A 05
	1	1	P059	2X34	2B 35	1A 14
	2	3	P067	2X34	2B 35	1A 15
	1	2	P081	2X34	2B 20	1A 17
	1	1	P082	2X41	2B 35	1A 17
	2	1	P084	2X02	2B 20	1A 18
	2-4	27	P085	2X37	2B 20	1A 18
	2	2	P086	2X41	2B 20	1A 18
	2	1	P087	2X43	2B 20	1A 18
	2	1	P088	2X42	2B 25	1A 18
24F	≤ 0.015	1	P001	2X01	2B 01	1A 01
	≤ 0.015	2	P024	2X01	2B 01	1A 04
25A	0.25	1	P021	2X07	2B 12	1A 02
	0.12	1	P035	2X13	2B 08	1A 04
25F	0.25	1	P034	2X08	2B 08	1A 04
28A	≤ 0.015	2	P008	2X02	2B 04	1A 01
28F	≤ 0.015	3	P030	2X02	2B 03	1A 04
29	≤ 0.015	1	P002	2X03	2B 01	1A 01
	0.03	1	P009	2X03	2B 04	1A 01
	0.5	1	P017	2X26	2B 22	1A 01
33B	0.03	2	P027	2X15	2B 01	1A 04
34	≤ 0.015	3	P024	2X01	2B 01	1A 04
	0.03	1	P027	2X15	2B 01	1A 04
	0.03	1	P031	2X02	2B 04	1A 04
	≤ 0.015	1	P047	2X01	2B 01	1A 06
	0.03	1	P050	2X10	2B 02	1A 07
NT	1	1	P101	2X33	2B 46	1A 25

Serotype 3 had the most PBPs substitution combinations, with a total of 13 groups. Except for the P047 combination, in which strains had PEN MICs ranging between 0.015-0.03 μg/ml, strains in the other 12 groups all had a PEN MIC of ≤0.015 μg/ml. The next serotype with the most PBPs substitution combinations was 23F with 11combinations. Save for the PEN MIC of 3 strains in group P038 and 1 strain in group P040 which were 0.5 μg/ml and ≤ 0.015 μg/ml, respectively, the PEN MIC of 42 strains in the other nine PBPs substitution combinations were between 1- 4 μg/ml. A total of 10, 8, 7, 7, 5, 5 PBPs substitution combinations were detected in serotypes 19F, 6B, 14, 19A, 6A, and 34, respectively. The number of the corresponding substitution combinations in other serotypes were ≤4. The strains of serotypes 1 (*n* = 5, P031), 2 (*n* = 1, P022), 4 (*n* = 1, P024), 7F (*n* = 2, P015), 8 (*n* = 4, P023), 12F (*n* = 4, P020), 17 (*n* = 1, P047), 17A (*n* = 1, P047), 18C (*n* = 1, P042), 28A (*n* = 2, P008), 28F (*n* = 3, P030) all had a PEN MIC ≤ 0.015 μg/ml and all strains within one serotype belonged to the same PBPs substitution combination. Serotypes 22F, 25F, and NT only had one strain in each group and PEN MICs were 0.5, 0.25, and 1 μg/ml respectively, corresponding to the unique substitution combination P037, P034, and P101, respectively.

## Discussion

In this study, 300 *S. pneumoniae* strains isolated from IPD were analyzed for serotype distribution, antimicrobial susceptibility and PBPs substitutions. To our knowledge, this is the largest multicenter study involving invasive *S. pneumoniae* strains in China, in terms of number and diversity of origin of the isolates studied. Based on the Quellung reaction, 40 different serotypes were detected amongst 299 typeable *S. pneumoniae* strains, with the five most common serotypes being 23F, 19A, 19F, 3, and 14. The serotype distribution in the current study is consistent with those from other studies in China, although the isolation rates varied (Zhao et al., 2013, 2020).

All strains were susceptible to ETP, LEV, LZD, and VA, which is consistent with most studies in our region and abroad (Zhanel et al., 2006; Zhao et al., 2013), (Zhou et al., 2016; Cai et al., 2018; Fu et al., 2019; Golden et al., 2019; Shi et al., 2019). AMC was the second most active antimicrobial drug with a non-susceptibility rate of 2.3%, which is significantly lower than that reported in the national data for 2006 – 2008 in which the non- susceptibility rate was 5.3%, and the one from Beijing for the period 2012 and 2017 (10.2%) (Zhanel et al., 2006; Shi et al., 2019). A meta-analysis of invasive *S. pneumoniae* strains from Chinese children (2000 to 2016) also showed a very high proportion of AMC resistance among the isolates at 16.1%, which may be due to the higher proportion of PCV7-related serotype strains in these studies of 60.8%, compared to 42.3% in the present study (Fu et al., 2019). It has been previously reported that invasive *S. pneumoniae* strains of PCV7-related serotypes are more resistant to AMC than non-PCV7 serotype strains (15.7% vs. 1.7, *P* = 0.013) (Liu et al., 2010).

PRSP accounted for 70.2% of CSF derived strains in this study, which is lower than in previous studies based on invasive *S. pneumoniae* strains from children only, ranging from 76.6 to 95.7%, but higher than that reported in isolates from IPD cases in both adults and children, at 51.5% (Xue et al., 2010; Zhou et al., 2016; Shi et al., 2019). No PRSP was detected in strains from non-CSF specimens, and PISP accounted for 4.5% of the isolates, which was higher than that reported in IPD isolates from both adults and children at 3.8% (Zhao et al., 2013), but significantly lower than reported in children isolates from Shenyang (2010 to 2014), in which the resistance rate was very high at 32.3% (Fu et al., 2019). According to the oral breakpoint interpretation for all strains studied, PISP and PRSP accounted for 23.0% and 44.7% of the isolates, respectively, which were higher than reported in the comprehensive meta-analysis study of invasive *S. pneumoniae* strains from children (oral break points PISP: 42.6%, PRSP: 32%) (Fu et al., 2019). Data from the SENTRY program of the global multi-center surveillance study (1997-2016) showed that the Asia-Pacific region had the lowest *S. pneumoniae* penicillin susceptibility rate (52.4%, oral breakpoint) but the highest multi-drug (49.8%) and pan-drug resistance (17.3%) rates, compared with North America, Europe, and Latin America. However, the SENTRY program did not include data from mainland China (Sader et al., 2019). Previous data based on 881 *S. pneumoniae* isolates from 23 teaching hospitals across China from 2011 to 2016, showed that PRSP accounted for 51.6% of the isolates (oral breakpoint), a rate which is slightly higher than in the present study, but only 11.6% of the isolates were considered invasive in that previous study (Zhao et al., 2017).

The resistance mechanism of *S. pneumoniae* to penicillin and other BLAs is mainly affected through the modification of PBPs (Hakenbeck et al., 2012). There are 6 different PBP types described in *S. pneumoniae*, including PBP1a, PBP1b, PBP2a, PBP2b, PBP2x and PBP3. They can be divided into three categories according to their molecular weights and functions; type A: high molecular weight PBP containing PBP1a, PBP1b and PBP2a with transglycosidase activity and transpeptidase activity; type B: high molecular weight PBPs containing PBP2b and PBP2x with transpeptidase activity; type C: low molecular weight PBPs containing PBP3 with carboxypeptidase activity (Zapun et al., 2008; Hakenbeck et al., 2012).

Three types of PBPs play a major role in BLAs resistance, namely PBP2x, PBP2b, and PBP1a (Zapun et al., 2008). Normally, BLAs can combine with serine at the PBPs active site to form serine esters, thereby inhibiting the synthesis of bacterial cell walls, and leading to bacterial death. When there is PBPs substitution, the reactivity for BLAs drugs is reduced resulting in limited effectiveness of the drugs and eventual development of drug resistance (Zapun et al., 2008). Studies have shown that substitutions in the active sites of PBP2x and PBP2b, and their adjacent sites only cause low levels of BLAs resistance (Laible et al., 1991; Grebe and Hakenbeck, 1996; Sifaoui et al., 1996). However, combination substitutions involving PBP1a and those of PBP2b and PBP2x, can cause high levels of BLAs resistance, but PBP1a substitution alone cannot cause an increase in BLAs resistance levels (Zerfass et al., 2009). Substitutions in the PBP2b active sites mainly cause bacteria to be resistant to penicillin, but have nothing to do with cephalosporin resistance. On the contrary, substitutions in the PBP2x active sites are mainly related to cephalosporin resistance, and can result in high-level resistance to third-generation cephalosporin when combined with PBP1a substitutions (Zapun et al., 2008; Hakenbeck et al., 2012).

Although many studies have been performed on PBPs, both domestically in Asia and abroad, there are limited studies on *S. pneumoniae* isolates from IPD in mainland China. Our study mainly focused on the gene substitutions of *pbp2x*, *pbp2b*, and *pbp1a* among 300 invasive *S. pneumoniae* strains and their relationship with PEN MIC and serotype. In this study, the variations in the PBPs gene active sites were highly diverse, and the number of substitutions were higher than those in previous studies (Zhou et al., 2016; Chu et al., 2018).

Among the invasive *S. pneumoniae* isolates studied, strains with PEN MIC ≤ 0.25 μg/ml had no PBPs active site substitutions, except for one strain of serotype 6A (MIC = 0.25 μg/ml). On the other hand, strains with PEN MIC ≥ 0.5 μg/ml all had PBPs active site substitutions, while strains with high PEN MIC levels (MIC ≥ 1 μg/ml) all had PBP1a substitutions, which were accompanied by PBP2x and PBP2b substitutions. Even if the antimicrobial susceptibility phenotype of invasive *S. pneumoniae* derived from non-CSF was interpreted as susceptible by the meningitis breakpoint, the strains still had substitutions in different PBPs active sites. Thus the PBPs substitution of the strains was mainly related to PEN MICs, and less influenced by specimen type and antibiotic breakpoints.

We analyzed a total of 338 amino acids from positions 259-596 of PBP2x, and 98 different substitutions at 73 amino acid positions were detected. According to the distribution of PEN MIC, the number of substitution sites of strains with PEN MIC ≤ 0.12 μg/ml were more than that of PBP1a and PBP2b, ranging from 10-32. With the increase in PEN MIC, the number of substitution sites increased also, and the strains with MIC = 0.5 μg/ml had the maximum number of substitutions. In the study of *Zhou et al.*, the average number of substitutions in PSSP strains was much higher for PBP2x than for PBP2b (29.26 vs. 8.22). Interestingly, they also found a significantly higher number of substitution sites in PRSP (65.55 ± 2.93) and PISP (63.37 ± 2.51) compared to PSSP (29.26 ± 27.88) (Zhou et al., 2016). Thus, it could be presumed that the MIC value of PEN was associated with the number of substitution sites in PBP2x. Specifically, common substitutions in PBP2x active sites included T338A, M339F, H394L, and L546V, which have also been reported in previous studies (Smith and Klugman, 1995; Zhou et al., 2016; Cai et al., 2018). In the present study, 181 strains had the T338A substitution and 39 strains also contained the M339F substitution. The PEN MIC of most strains was 2-4 μg/ml. The M339F substitution was first discovered in a highly resistant clinical strain in France, and then successively detected in the USA and Japan (Coffey et al., 1995; Asahi et al., 1999; Nagai et al., 2002). Previous crystal structure studies showed that the active center 337STMK of PBP2x which had the T338A and M339F double-site substitution was deformed. Furthermore, the serine active site Ser337 also presented another conformational change, resulting in reduction in the acylation efficiency of penicillin and cefotaxime by more than 20 times (Lu et al., 2001; Chesnel et al., 2003).

T338A and M339F substitutions have also been described in research reports from Taiwan, Japan, South Africa and other places, and were related to resistance in penicillin, amoxicillin and third-generation cephalosporins (Smith and Klugman, 1995; Du Plessis et al., 2002; Sanbongi et al., 2004; Davies et al., 2012; Liu et al., 2016; Zhou et al., 2016). Previous studies have shown that the L546V substitution is associated with high level resistance in BLAs, especially cephalosporins, but this substitution has also been reported in PSSP and cephalosporin-susceptible strains, suggesting that a single L546V substitution is not sufficient enough to cause high level resistance of BLAs (Nichol et al., 2002; Davies et al., 2012; Liu et al., 2016). Both crystal structure studies *in vitro* and transformation experiments *in vivo* have confirmed that R384G could affect the susceptibility of bacterial strains to penicillin and cefotaxime (Smith and Klugman, 2005; Maurer et al., 2008; Liu et al., 2016). A crystal structure study of PBP2x has found that the amino acid substitutions at position 371 and 384 affected the mobility of loop between amino acids 365-394 and were important for BLAs resistance (Carapito et al., 2006). In our study, strains with PBP2b sequences belonging to Group 2X27−2X43 all harbor the T338A substitution along with some other relevant substitutions, such as I371T, R384G, and M400T, but it is unknown which substitutions contribute to the resistance of the strains. Further experiments are needed to figure out the inner association between resistance and various substitutions. The Q552E substitution was located near the third catalytic motif 547KTG549 at the end of strand β3 loop, thus substitutions can led to an increase of the acylation efficiency for BLAs (Pernot et al., 2004; Zapun et al., 2008). In previous studies, Q552E was widely reported while in this study Q552V substitution was frequently detected in strains with PEN MIC ≤ 0.5 μg/ml. D567N substitution was previously detected in *S. pneumoniae* strains with high penicillin resistance in Taiwan, but has also been described in PSSP, which is consistent with the findings of this study (Liu et al., 2016).

A total of 372 amino acids from positions 305-676 of PBP2b were analyzed, and a total of 111 substitutions and 2 insertion variations at 88 amino acid sites were detected. Similar to PBP1a, the number of substitution sites in strains with PEN MIC ≤ 0.12 μg/ml was no more than 10. Furthermore, the number of substitution sites increased with MIC increase, but the most substitution sites were detected in strains with PEN MIC = 0.5 μg/ml. The two insertion variations YIW and YTW were found in two strains, located between 386SVVK and 443SSN in the active site of PBP2b, which have never been reported elsewhere. Previously, Japanese researchers reported that there was a duplication of the amino acid sequence WYT at the positions 429-431 between the amino acid positions 431-432 of PBP2b (Yamane et al., 1996). The insertion sequence was detected in 13 strains, of which 10 were of serotype 19, and three were serotype 6. In that study, the MIC of PEN and cefotaxime of *S. pneumoniae* strains carrying the above insertion variation (WYT amino acid sequence) were 0.125-2 μg/ml and 0.063-1 μg/ml, respectively (Yamane et al., 1996). In the present study, the two strains were of serotype 6B and NT and the PEN MIC were 0.5 μg/ml and 1 μg/ml respectively.

Previous studies have shown that the insertion of amino acid sequence between the conserved sequences of PBP2b in *Neisseria gonorrhoeae* reduced its affinity to PEN, further studies are needed to confirm its function in *S. pneumoniae* (Brannigan et al., 1990). Substitutions in the PBP2b active sites included T446A, T446S, and A619G, which have been reported in previous studies (Yamane et al., 1996; Sanbongi et al., 2004; Granger et al., 2005; Izdebski et al., 2008; Tian et al., 2008). *In vitro* experiments have shown that the affinity of T446A substitution strain to penicillin was 60% lower than that of the wild strain (Pagliero et al., 2004). However, researchers in the United States (USA), South Korea and Canada also found this substitution site in PSSP strains (Nagai et al., 2002; Baek et al., 2004; Granger et al., 2006), a finding which was also confirmed in one serotype 6A PSSP strain derived from blood (MIC = 0.25 μg/ml) in the present study. The A619G substitution has also been reported in strains in United States, Spain, Mexico, and other regions, and has been shown to be associated with high level amoxicillin resistance (Kosowska et al., 2004; Cafini et al., 2006). Other common substitutions identified in this study included E476G, Q438E, and L455I. Among them, Q438E and E476G have been reported in studies in Taiwan, France and Japan, and these substitutions were related increased levels of bacterial resistance to BLAs (Sanbongi et al., 2004; Chesnel et al., 2005; Liu et al., 2016). To the best of our knowledge, the L455I substitution is reported for the first time in this study.

A total of 344 amino acids from positions 312-655 of PBP1a were analyzed, and 105 different substitutions at 85 amino acid positions were detected. Based on PEN MIC distribution, isolates with MIC ≤ 0.12 μg/ml had no more than 10 substitution sites, and the substitution sites increased with increasing MIC. However, the maximum number of substitution sites was found in strains with MIC = 0.5 μg/ml. Substitutions in the PBP1a active sites, including T371A, T371S, and P432T, have been reported in multinational studies, and are related to high level resistance in penicillin and cephalosporin (Smith and Klugman, 1998; Liu et al., 2016; Zhou et al., 2016; Chu et al., 2018). *In vitro* site-directed mutagenesis experiments showed that T371A substitution reduces the acylation efficiency of PBP1a to cefotaxime and penicillin by 2.4 and 26 times, respectively, and TSQF574-577NTGY substitution reduces the acylation efficiency of PBP1a to cefotaxime and penicillin by 5.5 and 49 times, respectively (Job et al., 2008). *In vivo* transformation studies showed that only when the two substitutions exist at the same time, the resistance level of substitution strains to BLAs would increase significantly (Smith and Klugman, 1998; Job et al., 2008). In addition, the sites with high substitution rates in this study included S540T, N546G, A550P, T574N, S575T, Q576G, F577Y, and N609D, and part of these sites have been reported in some strains in Shenyang (Zhou et al., 2016).

Classification of isolates based on combination of substitution patterns in the three PBPs revealed a high degree of diversity among the isolates. Strains with the same serotype and PEN MIC exhibited different PBPs substitution combinations due to differences in the three PBPs substitution sites. For example, most strains of serotype 3 had a PEN MIC ≤ 0.015 μg/ml, but there were as many as 13 corresponding PBPs substitution combinations, although the difference between the different PBPs sites was small. Likewise, isolates with similar PBPs substitution combinations did not necessarily have the same PEN MIC levels, and this was reflected in serotypes 14, 19A, 19F, and 23F. The substitution combinations P100, P072, P070, and P085 were detected in isolates with PEN MICs between 2-4 μg/ml. A similar finding has also been reported previously, where five strains of serotype 23F with exactly the same PBPs substitutions and *murM* had PEN MICs ranging between 0.25-2 μg/ml (Chesnel et al., 2005).

In summary, we investigated the variations in *pbp2x*, *pbp2b*, and *pbp1a* genes, and serotype distribution of IPD *S. pneumoniae* isolates collected between 2010 and 2015 in China. We analyzed the serotype distribution, resistance to PEN and PBPs substitutions amongst these strains. There was a great diversity detected in PBPs substitutions patterns among the strains, suggesting that the PEN MIC level of *S. pneumoniae* may be affected by several other factors. Therefore, a comprehensive understanding of antibiotic resistance mechanism of *S. pneumoniae* needs to be further examined at the genomic level.

## Data Availability Statement

The original contributions presented in the study are included in the article/Supplementary Material, further inquiries can be directed to the corresponding author/s.

## Author Contributions

YX and ZL conceived and designed the work. MZ, LW, and ZW performed the experiments, data analysis, and wrote the manuscript. YW and TK revised the manuscript. All authors read and approved the final manuscript. All authors contributed to the article and approved the submitted version.

## Conflict of Interest

The authors declare that the research was conducted in the absence of any commercial or financial relationships that could be construed as a potential conflict of interest.

## Publisher’s Note

All claims expressed in this article are solely those of the authors and do not necessarily represent those of their affiliated organizations, or those of the publisher, the editors and the reviewers. Any product that may be evaluated in this article, or claim that may be made by its manufacturer, is not guaranteed or endorsed by the publisher.
