# Supplementary material for: Molecular Characterization of Penicillin-Binding Protein2x, 2b and 1a of Streptococcus pneumoniae Causing Invasive Pneumococcal Diseases in China: A Multicenter Study
Source: Front Microbiol. 2022 Mar 1;13:838790. doi: 10.3389/fmicb.2022.838790 (PMC8921733; doi:10.3389/fmicb.2022.838790)
Supplement: Supplementary file 1 [file Data_Sheet_1.docx]

**Table S1.** Geographic distribution of 300 invasive *S. pneumoniae* isolates.

| **Hospital** | **Province** | **No. of isolates** |
| --- | --- | --- |
| Children's Hospital of Shanghai | Shanghai | 30 |
| Shengjing Hospital of China Medical University | Liaoning | 25 |
| The Children's Hospital, Zhejiang University School of Medicine | Zhejiang | 22 |
| Affiliated Taihe Hospital of Hubei University of Medicine | Hubei | 18 |
| The Affiliated Hospital of Xuzhou Medical University | Jiangsu | 16 |
| Shandong Provincial Hospital | Shandong | 15 |
| Peking Union Medical College Hospital | Beijing | 15 |
| Wuxi People's Hospital | Jiangsu | 14 |
| Tangdu Hospital | Shanxi | 14 |
| Affiliated Hospital of North China University of Technology | Hebei | 14 |
| Renmin Hospital of Wuhan University | Hubei | 13 |
| The First Hospital of China Medical University | Liaoning | 10 |
| The First Hospital of Lanzhou University | Gansu | 9 |
| Zhejiang Provincial People's Hospital | Zhejiang | 9 |
| Haikou People's Hospital | Hainan | 9 |
| Dalian Municipal Central Hospital | Liaoning | 9 |
| Subei People's Hospital | Jiangsu | 9 |
| The First Affiliated Hospital of Anhui Medical University | Anhui | 9 |
| China-Japan Friendship Hospital | Beijing | 8 |
| The First People’s Hospital of Changzhou | Jiangsu | 7 |
| The Second Hospital of Hebei Medical University | Hebei | 7 |
| Sir Run Run Shaw Hospital, Zhejiang University School of Medicine | Zhejiang | 6 |
| Zhongda Hospital, Southeast University | Jiangsu | 5 |
| General Hospital of Shenyang Military Region | Liaoning | 3 |
| Tianjin Third Central Hospital | Tianjin | 2 |
| The Second Hospital of Tianjin Medical University | Tianjin | 1 |
| Shanghai East Hospital | Shanghai | 1 |
| **Total** |  | **300** |

**Table S2.** Association of penicillin susceptibility and PBP2x variations in 300 *S. pneumoniae* isolates compared to R6.

| 2X | Substitution site | ≤0.015 | 0.03 | 0.06 | 0.12 | 0.25 | 0.5 | 1 | 2 | 4 | Total | Substitution rate (%) |
| --- | --- | --- | --- | --- | --- | --- | --- | --- | --- | --- | --- | --- |
| 1 | I265F |  |  |  |  |  |  |  | 1 |  | 1 | 0.3 |
| 2 | I265L |  |  |  |  |  | 11 | 16 | 114 | 11 | 152 | 50.7 |
| 3 | P268T | 1 | 3 | 1 |  | 3 | 8 | 13 | 1 | 1 | 31 | 10.3 |
| 4 | D278N |  |  |  |  | 1 | 7 | 10 | 1 | 1 | 20 | 6.7 |
| 5 | A279S | 1 | 1 |  |  |  |  |  |  |  | 2 | 0.7 |
| 6 | A279V |  |  |  |  |  | 2 |  |  |  | 2 | 0.7 |
| 7 | Q281L |  |  |  |  |  | 11 | 22 | 118 | 11 | 162 | 54.0 |
| 8 | D311N |  |  |  |  |  | 11 | 22 | 118 | 13 | 164 | 54.7 |
| 9 | I318L |  |  |  |  | 1 | 7 | 10 | 1 |  | 19 | 6.3 |
| 10 | E320K |  |  |  |  | 1 | 9 | 11 | 1 |  | 22 | 7.3 |
| 11 | T338A |  |  |  |  |  | 17 | 32 | 119 | 13 | 181 | 60.3 |
| 12 | M339F |  |  |  |  |  | 1 | 2 | 32 | 4 | 39 | 13.0 |
| 13 | M343T | 1 | 3 | 1 |  | 3 | 19 | 30 | 87 | 9 | 153 | 51.0 |
| 14 | A346S |  |  |  |  |  | 11 | 31 | 119 | 13 | 174 | 58.0 |
| 15 | A347S |  |  |  |  |  | 13 | 31 | 119 | 13 | 176 | 58.7 |
| 16 | G355S |  |  |  |  |  | 13 | 32 | 119 | 13 | 177 | 59.0 |
| 17 | V358Y |  |  |  |  |  | 15 | 32 | 119 | 13 | 179 | 59.7 |
| 18 | N360D |  |  |  |  |  | 1 |  |  |  | 1 | 0.3 |
| 19 | L364F |  |  |  |  |  | 10 | 32 | 119 | 13 | 174 | 58.0 |
| 20 | I366M |  |  |  |  |  |  |  | 1 |  | 1 | 0.3 |
| 21 | I366V |  |  |  |  |  | 1 |  |  |  | 1 | 0.3 |
| 22 | A369V |  |  |  |  |  | 9 |  | 1 |  | 10 | 3.3 |
| 23 | I371T |  |  |  |  |  | 11 | 32 | 119 | 13 | 175 | 58.3 |
| 24 | E378A |  |  |  |  |  | 3 | 2 | 27 | 4 | 36 | 12.0 |
| 25 | E378D |  |  |  |  | 1 | 9 | 13 | 58 | 2 | 83 | 27.7 |
| 26 | E378G |  |  |  |  |  |  |  | 1 |  | 1 | 0.3 |
| 27 | G382S |  |  |  |  |  | 2 |  |  |  | 2 | 0.7 |
| 28 | G382T |  |  |  |  | 1 | 17 | 32 | 119 | 13 | 182 | 60.7 |
| 29 | R384G |  |  |  | 1 | 1 | 18 | 32 | 119 | 13 | 184 | 61.3 |
| 30 | M385T | 5 |  |  |  |  | 2 |  |  |  | 7 | 2.3 |
| 31 | S389L |  |  |  |  | 1 | 17 | 32 | 115 | 13 | 178 | 59.3 |
| 32 | H394L |  |  |  |  |  | 4 |  |  |  | 4 | 1.3 |
| 33 | M400T |  |  |  |  |  | 1 | 2 | 32 | 4 | 39 | 13.0 |
| 34 | T401S |  |  |  |  | 1 | 13 | 32 | 119 | 13 | 178 | 59.3 |
| 35 | A410T |  |  |  |  |  | 2 |  | 20 | 1 | 23 | 7.7 |
| 36 | N417K |  |  |  |  |  | 10 | 32 | 99 | 12 | 153 | 51.0 |
| 37 | N417S |  |  |  |  |  | 2 |  |  |  | 2 | 0.7 |
| 38 | A434S |  |  |  |  | 1 | 1 |  |  |  | 2 | 0.7 |
| 39 | N444S |  |  |  |  |  | 10 | 32 | 119 | 13 | 174 | 58.0 |
| 40 | Q447M | 1 |  |  |  | 1 | 4 |  |  |  | 6 | 2.0 |
| 41 | S449A |  |  |  |  | 1 | 2 |  |  |  | 3 | 1.0 |
| 42 | T459I |  |  |  |  |  |  |  | 1 |  | 1 | 0.3 |
| 43 | I462L | 3 | 1 |  |  |  | 15 | 32 | 119 | 13 | 183 | 61.0 |
| 44 | F465L | 1 |  |  |  |  |  |  |  |  | 1 | 0.3 |
| 45 | S481T |  |  |  |  |  | 1 |  |  |  | 1 | 0.3 |
| 46 | I483L | 3 | 1 |  |  |  | 5 |  |  |  | 9 | 3.0 |
| 47 | P486T |  |  |  |  |  | 11 | 32 | 119 | 13 | 175 | 58.3 |
| 48 | D488N | 13 | 1 |  |  | 2 | 12 | 32 | 119 | 13 | 192 | 64.0 |
| 49 | T490S | 3 | 1 |  |  |  | 14 | 32 | 119 | 13 | 182 | 60.7 |
| 50 | A491V | 3 | 1 |  |  |  | 15 | 32 | 119 | 13 | 183 | 61.0 |
| 51 | I498V |  |  |  | 1 |  | 1 |  |  |  | 2 | 0.7 |
| 52 | N501K |  |  |  |  |  | 1 |  | 27 | 7 | 35 | 11.7 |
| 53 | K505E |  |  |  |  |  | 1 |  | 27 | 7 | 35 | 11.7 |
| 54 | D506A | 3 | 1 |  |  |  | 3 |  |  |  | 7 | 2.3 |
| 55 | D506E |  |  |  |  |  | 10 | 32 | 92 | 6 | 140 | 46.7 |
| 56 | A507T |  |  |  |  |  |  |  | 27 | 7 | 34 | 11.3 |
| 57 | A507V |  |  |  |  |  |  |  | 1 |  | 1 | 0.3 |
| 58 | L510Q |  |  |  |  |  | 1 |  |  |  | 1 | 0.3 |
| 59 | L510S | 3 | 1 |  |  |  | 3 |  |  |  | 7 | 2.3 |
| 60 | L510T |  |  |  |  |  | 10 | 32 | 92 | 7 | 141 | 47.0 |
| 61 | T513D |  |  |  |  |  | 2 |  |  |  | 2 | 0.7 |
| 62 | T513E | 3 | 1 |  |  |  | 1 |  |  |  | 5 | 1.7 |
| 63 | T513N |  |  |  |  |  | 10 | 32 | 92 | 7 | 141 | 47.0 |
| 64 | N514H | 3 | 1 |  |  |  | 14 | 32 | 92 | 7 | 149 | 49.7 |
| 65 | N514Y |  |  |  |  |  | 2 |  |  |  | 2 | 0.7 |
| 66 | V516I |  |  |  | 1 | 1 | 16 | 32 | 119 | 13 | 182 | 60.7 |
| 67 | L517M | 3 | 1 |  |  |  | 3 |  |  |  | 7 | 2.3 |
| 68 | T520K | 1 |  |  |  |  |  |  |  |  | 1 | 0.3 |
| 69 | P522A | 1 | 3 | 1 |  | 2 |  |  |  |  | 7 | 2.3 |
| 70 | V523L |  |  |  |  |  | 11 | 32 | 119 | 13 | 175 | 58.3 |
| 71 | V523R |  |  |  |  |  | 1 |  |  |  | 1 | 0.3 |
| 72 | S531K |  |  |  |  |  | 1 |  |  |  | 1 | 0.3 |
| 73 | S531Q |  |  |  |  |  | 1 |  |  |  | 1 | 0.3 |
| 74 | S531Y |  |  |  |  |  | 10 | 32 | 119 | 13 | 174 | 58.0 |
| 75 | P535A |  |  |  |  |  | 1 |  |  |  | 1 | 0.3 |
| 76 | T536I |  |  |  |  |  | 11 | 32 | 119 | 13 | 175 | 58.3 |
| 77 | T536N | 3 | 1 |  |  |  | 3 |  |  |  | 7 | 2.3 |
| 78 | V537I |  |  |  |  |  | 11 | 32 | 119 | 13 | 175 | 58.3 |
| 79 | T538N | 4 | 1 |  |  |  | 4 |  |  |  | 9 | 3.0 |
| 80 | V544I |  |  |  |  |  | 1 |  |  |  | 1 | 0.3 |
| 81 | L546V |  |  |  |  |  | 11 | 32 | 119 | 13 | 175 | 58.3 |
| 82 | Q552E | 5 | 5 | 1 | 2 | 3 | 8 |  |  |  | 24 | 8.0 |
| 83 | K557R |  |  |  |  | 1 |  |  |  |  | 1 | 0.3 |
| 84 | V563T | 3 | 1 |  | 1 |  | 6 |  |  |  | 11 | 3.7 |
| 85 | L565E | 3 | 1 |  | 1 |  | 6 |  |  |  | 11 | 3.7 |
| 86 | L565S | 2 | 4 | 1 | 1 | 3 | 13 | 32 | 119 | 13 | 188 | 62.7 |
| 87 | L565T |  |  |  |  |  | 1 |  |  |  | 1 | 0.3 |
| 88 | L565V | 37 | 1 |  |  | 2 | 1 |  | 1 |  | 42 | 14.0 |
| 89 | D567N | 5 | 5 | 1 | 2 | 4 | 27 | 32 | 119 | 13 | 208 | 69.3 |
| 90 | Y568H |  |  |  |  |  | 1 |  |  |  | 1 | 0.3 |
| 91 | Y568N | 5 | 5 | 1 | 2 | 3 | 8 |  |  |  | 24 | 8.0 |
| 92 | A572D | 1 |  |  |  |  |  |  |  |  | 1 | 0.3 |
| 93 | A572V | 5 | 5 | 1 | 2 | 3 | 17 | 15 | 90 | 6 | 144 | 48.0 |
| 94 | S574A | 2 | 4 | 1 | 1 | 3 | 2 |  |  |  | 13 | 4.3 |
| 95 | S574T |  |  |  |  |  | 11 | 32 | 119 | 13 | 175 | 58.3 |
| 96 | S576H | 3 | 1 |  | 1 |  | 6 |  |  |  | 11 | 3.7 |
| 97 | S576N | 2 | 4 | 1 | 1 | 4 | 16 | 32 | 119 | 13 | 192 | 64.0 |
| 98 | Y595F |  |  |  |  |  | 1 | 2 | 32 | 4 | 39 | 13.0 |
|  | Total | 32 | 27 | 10 | 13 | 25 | 86 | 46 | 57 | 48 |  |  |

**Table S3.** Association of penicillin susceptibility and PBP2b variations in 300 *S. pneumoniae* isolates compared to R6.

| 2B | Substitution site | ≤0.015 | 0.03 | 0.06 | 0.12 | 0.25 | 0.5 | 1 | 2 | 4 | Total | Substitution rate (%) |
| --- | --- | --- | --- | --- | --- | --- | --- | --- | --- | --- | --- | --- |
| 1 | A315S |  |  |  |  |  |  | 2 | 3 |  | 5 | 1.7 |
| 2 | A315T |  |  |  | 1 | 1 |  |  |  |  | 2 | 0.7 |
| 3 | S319N | 4 |  |  |  |  |  |  |  |  | 4 | 1.3 |
| 4 | D321E | 1 |  |  |  |  |  |  |  |  | 1 | 0.3 |
| 5 | A322S |  |  |  |  |  |  | 2 | 3 |  | 5 | 1.7 |
| 6 | E333G |  |  |  |  |  | 10 | 28 | 118 | 13 | 169 | 56.3 |
| 7 | E333T |  |  |  |  |  |  | 2 | 3 |  | 5 | 1.7 |
| 8 | S357A |  |  |  |  |  |  | 2 | 3 |  | 5 | 1.7 |
| 9 | I361L |  |  |  |  |  | 6 | 23 | 59 | 11 | 99 | 33.0 |
| 10 | D364N |  |  |  |  |  |  | 2 | 3 |  | 5 | 1.7 |
| 11 | K366E |  |  |  |  |  |  | 2 | 3 |  | 5 | 1.7 |
| 12 | K366N |  |  |  |  |  | 1 | 4 | 54 | 7 | 66 | 22.0 |
| 13 | E369D |  |  |  |  |  | 1 | 2 | 27 | 3 | 33 | 11.0 |
| 14 | P372Q |  |  |  |  |  | 1 | 4 | 20 | 1 | 26 | 8.7 |
| 15 | V383I |  |  |  |  |  |  |  |  | 1 | 1 | 0.3 |
| 16 | Q406P |  |  |  |  |  |  |  | 1 |  | 1 | 0.3 |
| 17 | Q411H |  |  |  |  |  | 1 |  |  |  | 1 | 0.3 |
| 18 | S412P |  |  |  |  |  | 14 | 31 | 121 | 12 | 178 | 59.3 |
| 19 | S412T |  |  |  |  |  |  |  |  | 1 | 1 | 0.3 |
| 20 | V414F |  |  |  |  |  |  |  |  | 1 | 1 | 0.3 |
| 21 | A419S |  |  |  |  |  | 1 | 1 |  |  | 2 | 0.7 |
| 22 | N422Y |  |  |  |  |  | 13 | 24 | 121 | 12 | 170 | 56.7 |
| 23 | 424YIW |  |  |  |  |  |  | 1 |  |  | 1 | 0.3 |
| 24 | 424YTW |  |  |  |  |  | 1 |  |  |  | 1 | 0.3 |
| 25 | T426K |  |  |  |  |  | 13 | 31 | 121 | 13 | 178 | 59.3 |
| 26 | Q427A |  |  |  |  |  | 4 |  |  |  | 4 | 1.3 |
| 27 | Q427L |  |  |  |  |  | 13 | 24 | 121 | 12 | 170 | 56.7 |
| 28 | A428F |  |  |  |  |  | 4 |  |  |  | 4 | 1.3 |
| 29 | Y429S |  |  |  |  |  | 4 |  |  |  | 4 | 1.3 |
| 30 | G430R |  |  |  |  |  | 1 |  |  |  | 1 | 0.3 |
| 31 | G430V |  |  |  |  |  | 3 |  |  |  | 3 | 1.0 |
| 32 | S431P |  |  |  |  |  | 4 |  |  |  | 4 | 1.3 |
| 33 | F432M |  |  |  |  |  | 4 |  |  |  | 4 | 1.3 |
| 34 | V437I | 4 |  |  |  |  |  |  |  |  | 4 | 1.3 |
| 35 | Q438E |  |  |  |  |  | 21 | 31 | 121 | 12 | 185 | 61.7 |
| 36 | A439T |  |  |  |  |  | 1 |  |  |  | 1 | 0.3 |
| 37 | T446A |  |  |  |  | 1 | 25 | 31 | 121 | 12 | 190 | 63.3 |
| 38 | T446S |  |  |  |  |  | 1 | 1 |  | 1 | 3 | 1.0 |
| 39 | V449I |  |  |  |  |  |  |  |  | 1 | 1 | 0.3 |
| 40 | L455I |  |  |  |  |  | 20 | 30 | 121 | 12 | 183 | 61.0 |
| 41 | G467D | 3 |  |  |  |  |  |  |  |  | 3 | 1.0 |
| 42 | G467L |  |  |  |  |  | 8 |  |  |  | 8 | 2.7 |
| 43 | S469N |  |  |  |  |  | 8 |  |  |  | 8 | 2.7 |
| 44 | N470K |  |  |  |  |  | 1 |  |  |  | 1 | 0.3 |
| 45 | S473T |  |  |  |  |  | 12 | 23 | 121 | 12 | 168 | 56.0 |
| 46 | E476G |  |  |  |  | 1 | 27 | 32 | 121 | 13 | 194 | 64.7 |
| 47 | S480A |  |  |  |  |  | 12 | 23 | 121 | 12 | 168 | 56.0 |
| 48 | G483A |  |  |  |  |  | 8 |  |  |  | 8 | 2.7 |
| 49 | T489A |  |  |  |  | 1 | 20 | 23 | 121 | 12 | 177 | 59.0 |
| 50 | T489S |  |  |  |  |  | 7 | 9 |  | 1 | 17 | 5.7 |
| 51 | A490S |  |  |  |  |  | 8 |  |  |  | 8 | 2.7 |
| 52 | D497Y |  |  |  |  |  |  |  |  | 1 | 1 | 0.3 |
| 53 | F502L |  |  |  |  |  |  |  |  | 1 | 1 | 0.3 |
| 54 | V503I |  |  |  |  |  | 14 |  |  |  | 14 | 4.7 |
| 55 | E506D |  |  |  |  | 1 | 6 | 3 |  |  | 10 | 3.3 |
| 56 | S508N |  |  |  |  | 1 | 12 |  |  |  | 13 | 4.3 |
| 57 | Y512F |  |  |  |  | 1 | 4 | 7 |  |  | 12 | 4.0 |
| 58 | A510P | 3 |  |  |  |  |  |  |  |  | 3 | 1.0 |
| 59 | A516S |  |  |  |  |  | 1 | 6 | 39 | 1 | 47 | 15.7 |
| 60 | A516T |  |  |  |  |  |  |  |  | 1 | 1 | 0.3 |
| 61 | F520S | 1 |  |  |  |  |  |  |  |  | 1 | 0.3 |
| 62 | A533G |  |  |  |  |  | 1 |  |  |  | 1 | 0.3 |
| 63 | N538D | 3 |  |  |  | 3 | 25 | 25 | 79 | 12 | 147 | 49.0 |
| 64 | V542I |  |  |  |  |  | 1 |  |  |  | 1 | 0.3 |
| 65 | V542L |  |  |  |  |  |  | 2 |  |  | 2 | 0.7 |
| 66 | R545H |  |  |  |  |  | 1 | 2 |  |  | 3 | 1.0 |
| 67 | G552D |  |  |  |  |  |  | 2 |  |  | 2 | 0.7 |
| 68 | D555E |  |  |  |  |  | 1 |  |  |  | 1 | 0.3 |
| 69 | K556Q |  |  |  |  |  | 1 |  |  |  | 1 | 0.3 |
| 70 | D561E |  |  |  |  |  | 1 | 3 | 58 | 10 | 72 | 24.0 |
| 71 | D561N | 1 |  |  |  |  | 1 |  |  |  | 2 | 0.7 |
| 72 | Q565A |  |  |  |  |  | 1 | 3 | 58 | 10 | 72 | 24.0 |
| 73 | Q565S |  |  |  |  |  | 1 |  |  |  | 1 | 0.3 |
| 74 | L566I |  |  |  |  |  | 1 | 3 | 58 | 10 | 72 | 24.0 |
| 75 | L566V |  |  |  |  |  | 1 |  |  |  | 1 | 0.3 |
| 76 | Q567D |  |  |  |  |  | 1 | 3 | 58 | 10 | 72 | 24.0 |
| 77 | Q567E |  |  |  |  |  | 1 |  |  |  | 1 | 0.3 |
| 78 | P568S |  |  |  |  |  | 1 |  |  |  | 1 | 0.3 |
| 79 | P568T |  |  |  |  |  | 1 | 3 | 58 | 10 | 72 | 24.0 |
| 80 | T569I |  |  |  |  |  |  | 7 |  |  | 7 | 2.3 |
| 81 | T569K |  |  |  |  |  | 2 | 3 | 58 | 10 | 73 | 24.3 |
| 82 | M571I | 1 | 1 |  |  | 1 | 2 | 3 | 58 | 10 | 76 | 25.3 |
| 83 | V574I |  |  |  |  |  | 1 |  |  |  | 1 | 0.3 |
| 84 | D578E |  |  |  |  |  | 2 | 3 | 58 | 10 | 73 | 24.3 |
| 85 | M581V |  |  |  |  |  | 1 |  |  |  | 1 | 0.3 |
| 86 | S582A |  |  |  |  |  | 1 | 3 | 58 | 10 | 72 | 24.0 |
| 87 | I583V |  |  |  |  | 1 | 12 |  |  |  | 13 | 0.3 |
| 88 | H585Q |  |  |  |  |  | 1 |  |  |  | 1 | 24.3 |
| 89 | A592S |  |  |  |  |  | 1 | 3 | 58 | 11 | 73 | 0.3 |
| 90 | T595G |  |  |  |  |  | 1 |  |  |  | 1 | 0.3 |
| 91 | G597A |  |  |  |  |  | 1 |  |  |  | 1 | 11.7 |
| 92 | G597E | 30 | 3 |  | 1 | 1 |  |  |  |  | 35 | 24.0 |
| 93 | G597P |  |  |  |  |  |  | 3 | 58 | 11 | 72 | 24.0 |
| 94 | N606D |  |  |  |  |  |  | 3 | 58 | 11 | 72 | 3.3 |
| 95 | L609A |  |  |  |  |  | 5 | 4 | 1 |  | 10 | 0.7 |
| 96 | L609S |  |  |  |  |  | 2 |  |  |  | 2 | 24.0 |
| 97 | L609T |  |  |  |  |  |  | 3 | 58 | 11 | 72 | 24.0 |
| 98 | A619G |  |  |  |  |  |  | 3 | 58 | 11 | 72 | 1.3 |
| 99 | A624E |  |  |  |  |  | 3 | 1 |  |  | 4 | 28.3 |
| 100 | D625G |  |  |  |  |  | 8 | 7 | 59 | 11 | 85 | 0.3 |
| 101 | Q628D |  |  |  |  |  |  |  |  | 1 | 1 | 30.7 |
| 102 | Q628E |  |  |  |  | 1 | 15 | 7 | 59 | 10 | 92 | 0.3 |
| 103 | T630D |  |  |  |  |  |  |  | 1 |  | 1 | 27.7 |
| 104 | T630N |  |  |  |  |  | 8 | 7 | 58 | 10 | 83 | 0.3 |
| 105 | T630S |  |  |  |  |  |  |  |  | 1 | 1 | 24.0 |
| 106 | S640T |  |  |  |  |  |  | 3 | 58 | 11 | 72 | 24.0 |
| 107 | D641E |  |  |  |  |  |  | 3 | 58 | 11 | 72 | 24.0 |
| 108 | N659K |  |  |  |  |  |  | 3 | 58 | 11 | 72 | 24.0 |
| 109 | G660N |  |  |  |  |  |  | 3 | 58 | 11 | 72 | 24.0 |
| 110 | S664A |  |  |  |  |  |  | 3 | 58 | 11 | 72 | 25.7 |
| 111 | Q674N |  |  |  |  |  | 4 | 4 | 58 | 11 | 77 | 25.7 |
| 112 | K675Q |  |  |  |  |  | 4 | 4 | 58 | 11 | 77 | 25.7 |
| 113 | Y676H |  |  |  |  |  | 4 | 4 | 58 | 11 | 77 |  |
|  | Total | 10 | 2 | 0 | 2 | 12 | 75 | 61 | 52 | 54 |  |  |

**Table S4.** Association of penicillin susceptibility and PBP1a variations in 300 *S. pneumoniae* isolates compared to R6.

| 1A | Substitution site | ≤0.015 | 0.03 | 0.06 | 0.12 | 0.25 | 0.5 | 1 | 2 | 4 | Total | Substitution rate (%) |
| --- | --- | --- | --- | --- | --- | --- | --- | --- | --- | --- | --- | --- |
| 1 | D312N |  |  |  |  |  | 3 |  |  |  | 3 | 1.0 |
| 2 | T316S |  |  |  |  |  | 9 | 28 | 113 | 13 | 163 | 54.3 |
| 3 | D317E |  |  |  |  |  | 1 |  |  |  | 1 | 0.3 |
| 4 | E318Q |  |  |  |  |  | 9 | 28 | 113 | 13 | 163 | 54.3 |
| 5 | V320I |  |  |  |  |  |  | 1 |  |  | 1 | 0.3 |
| 6 | A321S |  |  |  |  |  | 9 | 28 | 113 | 13 | 163 | 54.3 |
| 7 | E326D |  |  |  |  |  | 9 | 28 | 113 | 13 | 163 | 54.3 |
| 8 | I333V |  |  |  |  |  | 9 | 29 | 121 | 13 | 172 | 57.3 |
| 9 | S337T |  |  |  |  |  | 3 |  |  |  | 3 | 1.0 |
| 10 | A347S |  |  |  |  |  | 1 | 2 |  |  | 3 | 1.0 |
| 11 | S351A |  |  |  |  |  | 9 | 29 | 121 | 13 | 172 | 57.3 |
| 12 | I358T |  |  |  |  |  | 9 | 29 | 121 | 13 | 172 | 57.3 |
| 13 | T371A |  |  |  |  |  | 4 | 8 | 40 | 1 | 53 | 17.7 |
| 14 | T371S |  |  |  |  |  | 6 | 23 | 81 | 12 | 122 | 40.7 |
| 15 | L382I |  |  |  |  |  | 4 | 6 | 40 | 1 | 51 | 17.0 |
| 16 | G385D |  |  |  |  |  | 1 | 2 |  |  | 3 | 1.0 |
| 17 | V386I | 16 | 1 |  |  |  | 1 | 2 |  |  | 20 | 6.7 |
| 18 | E388D | 88 | 8 | 1 | 2 | 7 | 28 | 32 | 121 | 13 | 300 | 100.0 |
| 19 | T392S |  |  |  |  |  | 6 | 16 | 81 | 12 | 115 | 38.3 |
| 20 | I393M |  |  |  |  |  | 4 | 15 | 40 | 1 | 60 | 20.0 |
| 21 | H395N |  |  |  |  |  | 4 | 15 | 40 | 1 | 60 | 20.0 |
| 22 | E397I |  |  |  |  |  | 4 | 15 | 40 | 1 | 60 | 20.0 |
| 23 | E397V |  |  |  |  |  | 6 | 16 | 81 | 12 | 115 | 38.3 |
| 24 | N405D |  |  |  |  |  | 11 | 16 | 74 | 9 | 110 | 36.7 |
| 25 | N405S |  |  |  |  |  | 4 | 15 | 47 | 4 | 70 | 23.3 |
| 26 | T406I |  |  |  |  |  | 3 |  |  |  | 3 | 1.0 |
| 27 | V408L |  |  |  |  |  | 6 | 16 | 81 | 12 | 115 | 38.3 |
| 28 | R413H |  |  |  |  |  | 5 | 14 | 81 | 12 | 112 | 37.3 |
| 29 | R413K |  |  |  |  |  | 1 | 2 |  |  | 3 | 1.0 |
| 30 | G414A |  |  |  |  |  | 4 | 15 | 40 | 1 | 60 | 20.0 |
| 31 | G414V |  |  |  |  |  | 6 | 16 | 81 | 12 | 115 | 38.3 |
| 32 | L421I |  |  |  |  |  | 6 | 16 | 81 | 12 | 115 | 38.3 |
| 33 | P432T |  |  |  |  |  | 10 | 31 | 121 | 13 | 175 | 58.3 |
| 34 | N443D |  |  |  |  |  | 10 | 31 | 121 | 13 | 175 | 58.3 |
| 35 | I459L |  |  |  |  |  | 3 |  |  |  | 3 | 1.0 |
| 36 | I459M |  |  |  |  |  | 9 | 31 | 121 | 13 | 174 | 58.0 |
| 37 | S462A |  |  |  |  |  | 10 | 31 | 121 | 13 | 175 | 58.3 |
| 38 | D473N |  |  |  |  |  | 12 | 22 | 121 | 13 | 168 | 56.0 |
| 39 | K474Q |  |  |  |  |  | 1 |  |  |  | 1 | 0.3 |
| 40 | K475H |  |  |  |  |  | 1 |  |  |  | 1 | 0.3 |
| 41 | K475Q |  |  |  |  |  | 3 | 7 | 40 | 1 | 51 | 17.0 |
| 42 | T495I |  |  |  |  |  | 11 | 18 | 90 | 12 | 131 | 43.7 |
| 43 | Y497H |  |  |  |  |  | 11 | 18 | 90 | 12 | 131 | 43.7 |
| 44 | H503N |  |  |  |  |  | 11 | 18 | 90 | 12 | 131 | 43.7 |
| 45 | V505I |  |  |  |  |  | 9 | 15 | 81 | 12 | 117 | 39.0 |
| 46 | E512K |  |  |  |  |  | 3 | 6 | 32 | 1 | 42 | 14.0 |
| 47 | E512S |  |  |  |  |  | 1 | 2 |  |  | 3 | 1.0 |
| 48 | E514Q | 5 |  |  |  |  |  |  |  |  | 5 | 1.7 |
| 49 | F515L |  |  |  |  |  | 1 |  |  |  | 1 | 0.3 |
| 50 | F515Y |  |  |  |  |  | 1 | 2 |  |  | 3 | 1.0 |
| 51 | S516A |  |  |  |  |  | 1 | 2 |  |  | 3 | 1.0 |
| 52 | N517D |  |  |  |  |  | 11 | 18 | 90 | 12 | 131 | 43.7 |
| 53 | V518A |  |  |  |  |  | 8 | 13 | 81 | 12 | 114 | 38.0 |
| 54 | V518P |  |  |  |  |  | 1 | 2 |  |  | 3 | 1.0 |
| 55 | T520I |  |  |  |  |  |  | 3 |  |  | 3 | 1.0 |
| 56 | D533E | 6 | 1 |  |  |  | 11 | 19 | 90 | 12 | 139 | 46.3 |
| 57 | S540A |  |  |  |  |  | 4 | 14 | 9 |  | 27 | 9.0 |
| 58 | S540T | 34 | 5 | 1 | 2 | 3 | 13 | 17 | 112 | 13 | 200 | 66.7 |
| 59 | S540V |  |  |  |  | 1 | 3 |  |  |  | 4 | 1.3 |
| 60 | T543I |  |  |  |  | 1 | 3 |  |  |  | 4 | 1.3 |
| 61 | N546G |  |  |  |  | 1 | 14 | 31 | 121 | 13 | 180 | 60.0 |
| 62 | A550P |  |  |  |  | 1 | 14 | 31 | 121 | 13 | 180 | 60.0 |
| 63 | P553A |  |  |  |  |  | 2 | 3 | 9 |  | 14 | 4.7 |
| 64 | E566D |  |  |  |  |  | 4 | 14 | 9 |  | 27 | 9.0 |
| 65 | I568V |  |  |  |  |  | 1 | 4 | 31 | 1 | 37 | 12.3 |
| 66 | N570K |  |  |  |  | 1 | 12 | 25 | 90 | 12 | 140 | 46.7 |
| 67 | H571Y |  |  |  |  | 1 | 10 | 15 | 81 | 12 | 119 | 39.7 |
| 68 | T574N |  |  |  |  | 1 | 14 | 31 | 121 | 13 | 180 | 60.0 |
| 69 | S575T |  |  |  |  | 1 | 14 | 31 | 121 | 13 | 180 | 60.0 |
| 70 | Q576G |  |  |  |  | 1 | 14 | 31 | 121 | 13 | 180 | 60.0 |
| 71 | F577Y |  |  |  |  | 1 | 14 | 31 | 121 | 13 | 180 | 60.0 |
| 72 | L583M |  |  |  |  | 1 | 12 | 31 | 121 | 13 | 178 | 59.3 |
| 73 | A585V |  |  |  |  | 1 | 12 | 31 | 121 | 13 | 178 | 59.3 |
| 74 | S592A |  |  |  |  | 1 | 1 |  |  |  | 2 | 0.7 |
| 75 | L606I |  |  |  |  |  | 11 | 31 | 121 | 13 | 176 | 58.7 |
| 76 | V607I |  |  |  |  |  | 1 | 1 | 20 | 1 | 23 | 7.7 |
| 77 | N609D |  |  |  |  | 1 | 14 | 31 | 121 | 13 | 180 | 60.0 |
| 78 | L611F |  |  |  |  |  | 11 | 31 | 121 | 13 | 176 | 58.7 |
| 79 | T612L |  |  |  |  |  | 10 | 29 | 121 | 13 | 173 | 57.7 |
| 80 | T612Y |  |  |  |  |  | 1 | 2 |  |  | 3 | 1.0 |
| 81 | A615G |  |  |  |  |  |  | 1 | 20 | 1 | 22 | 7.3 |
| 82 | K616R |  |  |  |  |  |  | 1 | 20 | 1 | 22 | 7.3 |
| 83 | M622I |  |  |  |  |  | 4 | 14 | 40 | 1 | 59 | 19.7 |
| 84 | T623S |  |  |  |  |  | 1 | 9 |  |  | 10 | 3.3 |
| 85 | G628D |  |  |  |  |  | 5 | 17 | 60 | 2 | 84 | 28.0 |
| 86 | S629D |  |  |  |  |  | 1 | 9 |  |  | 10 | 3.3 |
| 87 | S629G |  |  |  |  | 1 |  |  |  |  | 1 | 0.3 |
| 88 | S629N |  |  |  |  |  | 1 | 3 | 20 | 1 | 25 | 8.3 |
| 89 | S629R | 4 |  |  |  |  |  |  |  |  | 4 | 1.3 |
| 90 | S629T |  |  |  |  |  | 3 | 5 | 40 | 1 | 49 | 16.3 |
| 91 | N630H |  |  |  |  |  | 3 | 6 | 60 | 2 | 71 | 23.7 |
| 92 | N630Q |  |  |  |  |  | 1 | 9 |  |  | 10 | 3.3 |
| 93 | E632G |  |  |  |  |  | 2 | 12 | 20 | 1 | 35 | 11.7 |
| 94 | N635T |  |  |  |  |  | 5 | 17 | 60 | 2 | 84 | 28.0 |
| 95 | I636M |  |  |  |  |  | 5 | 17 | 60 | 2 | 84 | 28.0 |
| 96 | I636V |  |  |  |  |  | 4 |  |  |  | 4 | 1.3 |
| 97 | E638D |  |  |  |  |  | 6 | 14 | 40 | 1 | 61 | 20.3 |
| 98 | L640I |  |  |  |  |  | 2 |  |  |  | 2 | 0.7 |
| 99 | Y641F |  |  |  |  |  | 4 | 14 | 40 | 1 | 59 | 19.7 |
| 100 | N643S |  |  |  |  |  | 1 | 3 | 20 | 1 | 25 | 8.3 |
| 101 | E645Q |  |  |  |  |  | 1 |  |  |  | 1 | 0.3 |
| 102 | K649Q |  |  |  |  |  | 1 | 1 |  |  | 2 | 0.7 |
| 103 | N650K |  |  |  |  |  | 1 | 2 |  |  | 3 | 1.0 |
| 104 | S654N |  |  |  |  |  |  | 1 |  |  | 1 | 0.3 |
| 105 | S654P |  |  |  |  |  | 2 | 2 | 31 | 1 | 36 | 12.0 |
|  | Total | 6 | 4 | 2 | 2 | 17 | 97 | 88 | 71 | 68 |  |  |
